# Reconstructing pathogen-specific antibody binding epitopes and age-dependent immune signatures from proteomic-scale peptide libraries

**DOI:** 10.1126/sciadv.aec5478

**Published:** 2026-08-19

**Authors:** Everlyn Kamau, Nikolina Walas, Minlu Zhang, Kathy Kamath, Jack Reifert, John Shon, Shahjahan Ali, Md Ziaur Rahman, Abul K. Shoab, Syeda L. Famida, Salma Akther, Md Saheen Hossen, Palash Mutsuddi, Mahbubur Rahman, Jessica A. Grembi, Andrew N. Mertens, Richelle C. Charles, Daniel T. Leung, Stephen Luby, Audrie Lin, Benjamin F. Arnold

**Affiliations:** ^1^ Francis I. Proctor Foundation, University of California, San Francisco, San Francisco, CA, USA.; ^2^ School of Public Health, University of California, Berkeley, Berkeley, CA, USA.; ^3^ Serimmune Inc., Goleta, CA, USA.; ^4^ Department of Epidemiology, Colorado School of Public Health, Aurora, CO, USA.; ^5^ Environmental Health and WASH, Health System and Population Studies Division, International Centre for Diarrhoeal Disease Research, Dhaka, Bangladesh.; ^6^ Global Health and Migration Unit, Department of Women’s and Children’s Health, Uppsala University, Uppsala, Sweden.; ^7^ Department of Veterinary and Biomedical Sciences, The Pennsylvania State University, University Park, PA, USA.; ^8^ One Health Microbiome Center, Huck Life Sciences Institute, The Pennsylvania State University, University Park, PA, USA.; ^9^ Division of Epidemiology, University of California, Berkeley, Berkeley, CA, USA.; ^10^ Massachusetts General Hospital, Harvard Medical School, Harvard T.H. Chan School of Public Health, Boston, MA, USA.; ^11^ Division of Infectious Diseases, Department of Internal Medicine, University of Utah, Salt Lake City, UT, USA.; ^12^ Division of Infectious Diseases and Geographic Medicine, Stanford University, Stanford, CA, USA.; ^13^ Department of Microbiology and Environmental Toxicology, University of California, Santa Cruz, Santa Cruz, CA, USA.; ^14^ Department of Ophthalmology, University of California, San Francisco, San Francisco, CA, USA.; ^15^ Institute for Global Health Sciences, University of California, San Francisco, San Francisco, CA, USA.

## Abstract

High-density peptide arrays are useful for mapping linear antibody epitopes and resolution of antibody specificity in a single-assay platform. Here, we used peptide library screening to evaluate magnitude and breadth of humoral responses to enteric pathogens in the context of public health interventions. We characterized the epitope landscape to known immunogenic proteins to several viruses, bacteria, and parasites and used that to infer immunological profiles at age 3, 14, and 28 months. The peptide libraries detected immune signatures better for viruses compared with bacteria and parasites and captured distinct dynamics of protein-level variation in immune signatures over time. We found limited sensitivity for bacterial and protozoan pathogens whose humoral responses may depend more on conformational or natively modified epitopes than linear-peptide binding. Unbiased peptide libraries have potential utility for broad analysis of antibody responses in integrated serosurveillance, but our results reveal pathogen-specific limitations where peptide-based serology would be less predictive of true infection status and where it would provide greater surveillance value. Beyond assay performance, peptide arrays are useful for studying epitope architecture, and a practical implication would require augmentation with alternative antigen formats (whole-protein or folded antigens) in a complementary hybrid approach.

## INTRODUCTION

Diarrheal disease accounts for substantial childhood morbidity and mortality with more than half a million estimated deaths and more than four episodes per child per year among children younger than 5 years [Global Burden of Diseases, Injuries, and Risk Factors Study 2017; (*1*–*3*)]. Viral, bacterial, and parasitic enteropathogens, including enterotoxigenic *Escherichia coli*, rotavirus, *Shigella* spp., *Campylobacter* spp., norovirus, astrovirus, and *Cryptosporidium* spp., are leading causes of diarrheal illness and diarrheal-associated deaths in children living in low- and middle-income countries. In these settings, enteropathogen exposure is frequent with the primary window of infection occurring among those less than 3 years of age (*4*–*6*).

When used to study population-level transmission, serology or antibody-based measurements provide useful endpoints in intervention studies or randomized control trial designs with infrequent sampling or without continuous surveillance (*7*). This is because the immunologic signal detects infections that begin and resolve between sampling points. Antibody levels integrate exposure over time and embed information about infections in the intervals between measurements, therefore complementing pathogen detection to provide a more complete history of infection.

Phage and bacterial display assays present large libraries of potential peptide epitopes in tiled arrays to facilitate comprehensive mapping of all potential antibody recognition sites. This offers an advantage over traditional and other high-throughput serological techniques such as enzyme-linked immunosorbent assay (ELISA), lateral flow assays, and multiplex bead assays, which cannot readily or systematically achieve similar level of mapping (*8*, *9*). These highly multiplexed assays leverage high-throughput sequencing to profile antibody repertoires against entire pathogen proteomes without purifying the target proteins or analyzing each peptide individually (*8*, *10*), and individual- and group-specific serological profiles can be defined comparatively between those with and without disease or with and without infection (*11*–*13*). Random peptides can sometime mimic linear and discontinuous antigenic epitopes useful for determining minimal sequence for antibody binding (*14*). However, the drawback of the universality of the random peptide libraries is that their construction creates a possibility of displaying surface peptides not naturally present within an antigen and the phage clones may be of low affinity for the target molecule (*14*).

Here, we used a random bacterial display peptide library to determine serological profiles of various target enteric pathogens in the context of a household water, sanitation, and hygiene (WASH) and nutritional (N) intervention. The random peptide library allowed for tiling against arbitrary proteomes, whereas most phage display libraries would typically require selection of organisms before library construction. The opportunity to test for immune response to a large set of proteins and to reinterrogate results as previously unidentified proteins are discovered could be a major opportunity for valuable samples collected in randomized trials. To our knowledge, bacterial display assays have not been evaluated for their potential utility in randomized controlled trials of interventions that could affect several pathogens simultaneously.

The WASH Benefits cluster randomized trial in rural Bangladesh was conducted to assess, among other questions, whether improved WASH delivered to household compounds reduced diarrhea in a birth cohort compared with standard practices (*15*). While the primary outcomes of the Bangladesh study were caregiver-reported diarrhea and growth, we will report here results from serologic testing of samples in the same cohort. Our objective was to use protein array screening with the Bangladesh trial to detect differences or dynamic changes in immunoglobulin G (IgG) responses to known immunogenic proteins between intervention and control groups. We therefore hypothesized that (i) the WASH + N intervention would decrease enteric infections, leading to lower enteric pathogen immune responses compared with the control group; (ii) and that if children in the intervention group experienced fewer infections, then they could exhibit narrower breadth of IgG response at the epitope level compared with the control group who may have been infected multiple times by the same pathogen, with IgG providing a more integrated measure of cumulative exposure compared with a single stool sample. Conversely, if repeated infections led to a higher specificity of antibody response, then the control group may exhibit a narrower breadth (fewer IgG epitope hits).

## MATERIALS AND METHODS

### Ethics statement

Participants provided written, informed consent before enrollment and before specimen collection. The protocol of the original study (Clinical Trial Registration NCT01590095) was approved by Ethical Review Committees at the International Centre for Diarrheal Disease Research, Bangladesh (PR-11063); the Committee for the Protection of Human Subjects at University of California, Berkeley (2011-09-3652); the Institutional Review Board at Stanford University (25863); and the University of California, San Francisco (22-36722).

### Study design and eligibility

The WASH Benefits Bangladesh cluster-randomized trial was conducted in rural villages in Gazipur, Kishoreganj, Mymensingh, and Tangail districts and designed to generate evidence about the impact of sanitation, water quality, handwashing, and nutrition interventions on growth and development in early childhood (*16*). The trial enrolled 5551 pregnant women and evaluated outcomes at 1 year and 2 years of follow-up including caregiver-reported diarrhea in the past 7 days among children who were in utero or younger than 3 years at enrollment (*15*). The trial had seven arms: chlorinated drinking water (water); upgraded sanitation (sanitation); promotion of handwashing with soap (handwashing); combined water, sanitation, and handwashing; counselling on appropriate child nutrition plus lipid-based nutrient supplements (nutrition); combined water, sanitation, handwashing, and nutrition; and control (data collection only). The primary trial outcomes were caregiver-reported diarrhea in the past 7 days among children who were in utero or younger than 3 years at enrollment and linear growth (length-for-age *z*-score among children born to enrolled pregnant women) (*15*). The trial reported a 7-day diarrhea prevalence of 3.5% among index children and children under 3 years at enrollment who received combined WASH and nutrition (WASH + N), lower compared to a prevalence of 5.7% in the control group (*15*). Nutritional strategies including supplementation have been suggested to increase resistance to infections and are recommended for improving public health (*17*, *18*).

Here, we analyzed venous blood samples collected longitudinally in the trial at the median ages of 3, 14, and 28 months in a substudy from the control and combined WASH + N (“intervention”) groups to measure the effect of the combined intervention on immune responses among children (*19*) (see the Supplementary Materials for details on timeline of enrollment and the data collected). Sample collection occurred in 2013 and 2014. Our analysis included children in the two groups with blood samples collected at three follow-up visits: “visit 1,” median age 3 months; “visit 2,” median age 14 months; and “visit 3,” median age 28 months.

We applied two restrictions to improve balance between the two arms with respect to measurement dates and age at each visit. First, we excluded children whose first measurement was taken between August and November 2013 to improve comparability between arms in sampling timing with respect to calendar date and season, because measurement in control clusters was paused during that period due to political instability in Bangladesh. Second, we limited the cohort to children who were between 28 and 200 days old at visit 1 and between 365 and 548 days (1 to 1.5 years old) at visit 2. Conservatively assuming 50% seroprevalence in the control group at age 14 months, we determined that a sample size of 60 children per group would have at least 80% power to detect an absolute reduction of 25% seroprevalence (from 50 to 25%) with a two-sided α=0.05 using a standard equation for comparison of two proportions. Power for the expected quantitative outcomes could be higher, but, due to the novelty of the outcome measurement, there were no available data to inform power calculations. Therefore, among 147 children who met the inclusion and exclusion criteria, we selected a random sample of 120 children (60 per group, 360 total samples across time points) for analysis (fig. S1). Child age and sex were well balanced between groups, with slightly more female children and slightly older measurement ages at visits 1 and 2 in the control group (table S1).

### Serum antibody profiling using bacterial display libraries

#### Library preparation and selection

**Fig. 1.**
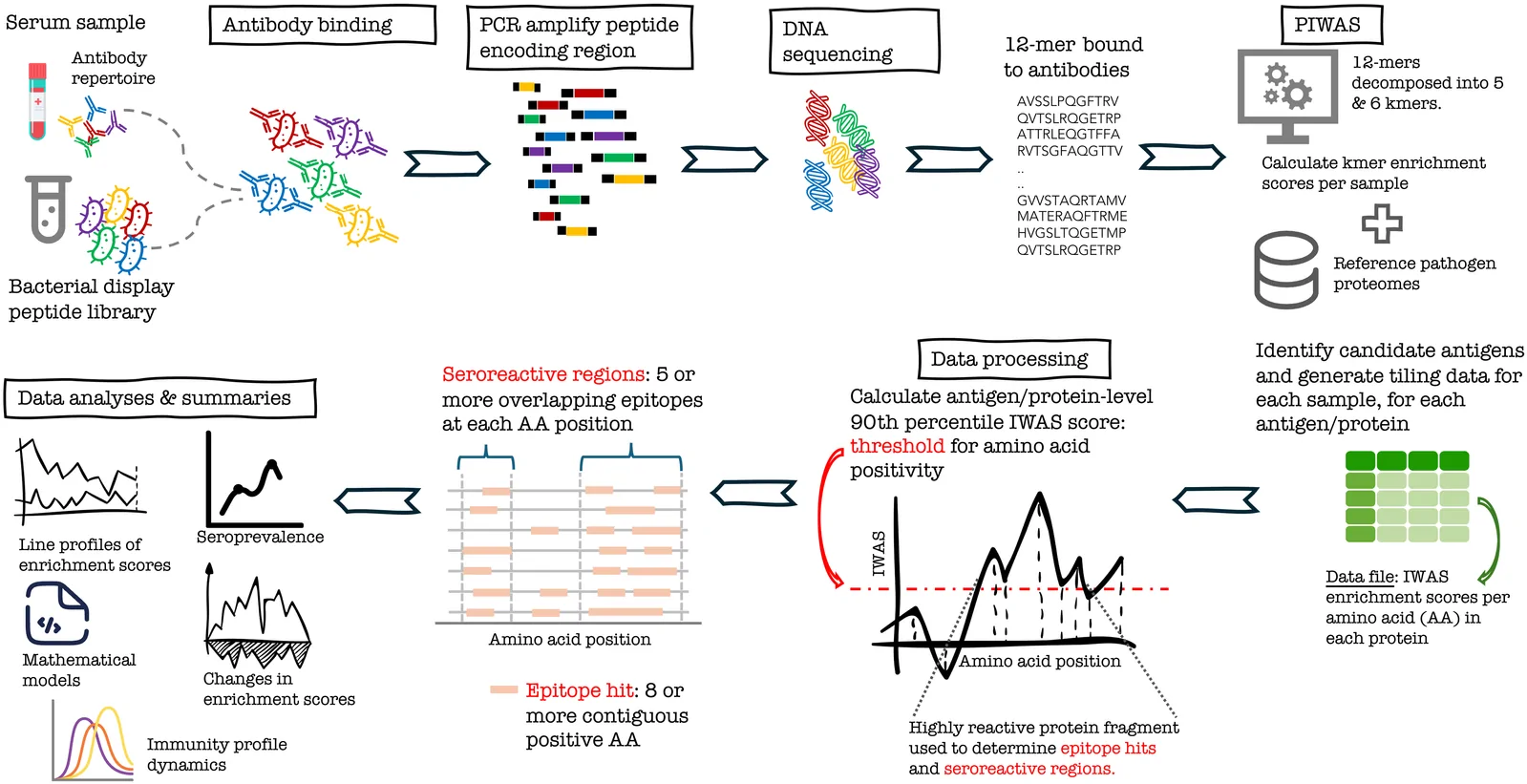
Sample processing and bioinformatic analysis. In the serum epitope repertoire analysis (SERA), serum samples were mixed with a fully random 12-mer peptide library expressed on the surface of *E. coli* bacteria, followed by isolation of the bacteria-antibody complexes using magnetic beads. Sequencing data from each sample were obtained and then processed to generate a nonredundant peptide list of antibody binding epitopes using the protein-based immunome-wide association study (PIWAS) algorithm. Each reconstructed 12-mer sequence was decomposed into 5- and 6-*k*-mer components, and log enrichments of these shorter *k*-mers were calculated at each amino acid position along a protein. We then determined epitope hits, and seroreactive regions were based on the enrichment scores. AA, amino acids; IWAS, immunome-wide association score.

Detection of the antibody repertoire composition was done using the serum epitope repertoire analysis (SERA) method published previously (Fig. 1) (*20*, *21*). Briefly, *E. coli* expressing random 12-nucleotide oligomer peptide display libraries were induced for peptide expression, resuspended in 15% glycerol/phosphate-buffered saline, and aliquoted in 96-well deep-well plates at 10-fold the library diversity in each well (8 × 10^10^ cells per well). Library selection was performed by incubating serum to a final 1:25 dilution and the antibody-bound bacterial clones captured using Protein A/G Sera-Mag SpeedBeads (GE Life Sciences, 17152104010350) (6.25% the beads’ stock concentration). The selected bacterial pools were resuspended in growth medium and incubated overnight at 37°C with shaking at 300 rpm for clone propagation (*20*, *21*).

#### PCR amplification and sequencing

Plasmids were isolated for the selected bacteria and the genomic regions encoding the random peptides amplified with polymerase chain reaction (PCR). Sample-specific indexing primers were used to incorporate adaptors and indices for barcoding individual samples and to enable sample pooling for Next Generation Sequencing (NGS) sequencing and data demultiplexing after sequencing (*20*, *21*). Sequencing was done according to specifications of the Illumina NextSeq 500 using a High Output v2, 75 cycle kit (Illumina, FC-404-2005).

#### Data preprocessing using the PIWAS method

For each sample, ∼750,000 to 3.6 million unique 12-mer sequences were obtained from the SERA assay and decomposed into constituent 5- and 6-mers to allow for identification of enriched signal and antibody selection in serum, i.e., increase the resolution of the exact binding amino acid positions of antibodies (*12*). We used the published protein-based immunome-wide association study (PIWAS) (*22*) to detect enriched IgG signal in target pathogen proteomes (table S2). IgG enrichment scores for each *k-*mer were calculated by dividing the number of unique 12-mers containing the *k-*mer by the number of expected *k-*mer reads for the sample, based on amino acid position composition of the sample. For each sample, the 5- and 6-mers were tiled against proteomes or protein sequences of interest. A tiling score was calculated at each amino acid on a protein based on the log enrichment of the corresponding 5- or 6-mer enrichment scores, with a smoothing window size of five 5-mers and five 6-mers. That is, a PIWAS value at each amino acid position along a protein sequence represents an averaged score within a five–amino acid frame using the tiling *z-*scores of 5-mers and 6-mers spanning the sequence and is, therefore, a smoothed antibody-binding intensity map along the protein sequence. Each amino acid’s tiling score thus reflects the magnitude of antibody signal at that position. As a final step, we calculated normalized *k-*mer enrichment values per amino acid position by normalizing the *z*-scores to a control population (healthy or unaffected individuals) as described in (*23*). The normalized *z*-scores are hereafter referred to as IgG enrichment values and represent antibody binding intensity.

SERA assay sensitivity and specificity are embedded in the PIWAS value calculations through comparisons of immunological signals of cases versus controls serum. Sensitivity for each protein is addressed through calculation of the outlier sum statistic, a *z*-score of permutations of enrichment values of cases and controls, which can further be translated into significance of the antigenic signal (*P* value) (*22*). Specificity is similarly addressed by normalizing K-mer enrichment values to those of a control population, thereby filtering out background signals. In addition, before normalizing, the K-mer enrichment calculation corrects for amino acid composition bias by calculating the ratio of the observed to expected counts of each K-mer within a given sample (*22*). In the Supplementary Materials (figs. S2 and S3), we show example alignments of individual-level normalized *z*-scores per amino acid position.

### Analysis of IgG enrichment values

#### Identification of linear protein-wide epitopes and seroreactive regions

Using the IgG enrichment values calculated above, an epitope in a protein was then defined as eight or more consecutive amino acid with a value above the 90th percentile enrichment value for that protein (see Fig. 1 for illustration and the Supplementary Materials for real example data). Higher IgG enrichment values indicate that antibodies bind the epitope or protein fragment more than would happen by chance. We chose a conservative minimum length of eight as it has been suggested that linear stretches in epitopes are characteristically seven amino acids in length and that antibody-binding energy is typically derived to about six residues (*24*–*26*). We then defined a seroreactive region, or an immunodominant region, as a contiguous protein fragment of high reactivity characterized by ≥5 overlapping epitopes at each amino acid position in that fragment. The relative start of a seroreactive region was the first amino acid position of the overlapping epitopes, after sorting the epitopes by their start amino acid residue. Accordingly, the relative end location was the last amino acid position of the overlapping epitopes sorted by length (see Fig. 1 for illustration and the Supplementary Materials for real example data). Therefore, the minimum length of a seroreactive region would be eight–amino acid residues consistent with a stack of ≥5 overlapping epitopes. An occurrence of <5 overlapping epitopes at an amino acid position introduced a gap between seroreactive regions (see the Supplementary Materials). We limited protein-level summaries to seroreactive regions to focus inference on protein regions with maximum signal or enhanced reactivity while ignoring infrequent epitope hits in the rest of the protein that could occur due to chance.

#### Definition of seropositivity, breadth, and magnitude

A sample was seropositive to a protein and, by extension, to a pathogen, if it had two or more epitope hits in the identified seroreactive regions. That is, after filtering for seroreactive regions only, seropositivity for a pathogen was defined as having at least two epitope hits across any of the queried proteins belonging to that pathogen. For example, a sample was seropositive for rotavirus if it had two or more epitope hits in seroreactive regions of the VP4 protein alone or collectively in seroreactive regions in VP4, VP7, and VP6 proteins. We chose two epitopes as the biologically meaningful minimum evidence for IgG binding, with the conceptual understanding that an IgG molecule has two Fab arms and can bind or recognize at least two epitopes simultaneously. We further restricted the putative binding to seroreactive regions, which we considered more likely to represent true antibody reactivity sites and reduces the likelihood that seropositivity is driven by a single isolated or nonspecific peptide signal. We note that there are no universal structural or diagnostic thresholds with peptide arrays and acknowledge that higher thresholds would have been more specific but not necessarily structurally required. We recognize that this could affect the inferred assay-derived serostatus and inferences of seroprevalence.

The number of epitope hits was used as a proxy for breadth of immune response. For each serum sample, and, for each evaluated pathogen protein, we defined breadth as “the number of epitopes located in seroreactive regions” and magnitude as “the average IgG signal intensity in the seroreactive regions.” We summarized breadth at each visit to assess population-level seroprevalence for various pathogens as described and shown in the Supplementary Materials and then narrowed our focus to five enteropathogens: (i) norovirus, (ii) *Helicobacter pylori*, (iii) rotavirus, (iv) adenovirus (HAdV), and (v) heat-stable toxin producing enterotoxigenic *E. coli* (ETEC-ST). From an initial assessment, seroprevalence of these enteropathogens broadly aligned with previous epidemiology in similar populations, whereas little or no IgG enrichment was detected to immunogenic proteins from *Campylobacter* spp., *Cryptosporidium* spp., *Salmonella enterica* serogroup Typhi, *Giardia* spp., *Vibrio cholerae*, *Strongyloides stercoralis*, heat-labile toxin producing enterotoxigenic *E. coli* (see the Supplementary Materials). Additionally, we included (vii) measles virus and (viii) poliovirus as to assess detection of IgG responses given that the age of the study cohort intersected with childhood immunization age.

### Comparison of method performance with Luminex multiplex bead assay

For the same samples, we compared seropositivity assignment using the bacteria peptide arrays (SERA assay) with data of IgG antibody responses measured using a custom in-house multiplex bead assay on the Luminex platform (see the Supplementary Materials). The multiplex bead assay had been assembled with a list of antigens targeting some of the pathogens described here and measured the median fluorescence intensity. We compared seropositivity for measles virus and norovirus, *Cryptosporidium* spp., *Campylobacter* spp., *S. enterica* serogroup Typhi, and *Giardia* sp. as they had been tested for IgG in both peptide arrays and multiplex assay.

### Assessment of positive and negative controls using previously developed immune repertoires

We evaluated the ability of the peptide arrays to examine the expected IgG responses to Epstein-Barr virus (EBV) and severe acute respiratory syndrome coronavirus 2 (SARS-CoV-2) as control pathogens with known historical exposure status. EBV is a ubiquitous human pathogen and, therefore, highly prevalent (positive control) and study children would not have experienced SARS-CoV-2 infections (negative control) since the study concluded in 2015. The SERA assay and PIWAS analysis (protein motif panels) used in this study were sensitive and specific for EBV and SARS-CoV-2 and were previously validated for profiling immune responses to these two pathogens (*27*). For EBV (latent or chronic infection), the SERA assay determined 62.5% seropositivity. For SARS-CoV-2, the assay determined 3% seropositivity.

### Statistical analyses

Statistical analyses were prespecified (https://osf.io/u92pc/). Children were analyzed according to their randomized group (intention to treat). The primary comparisons between the intervention and control groups focused on measurements in children ages 6 months or older while accounting for repeated measures within each child. We excluded measurements at ages <6 months from the between-group comparisons because of the likely contributions of maternal IgG (prespecified). Secondary analyses compared treatment groups at ages 14 and 28 months separately to examine age-dependent differences in the intervention effect. For quantitative comparisons, we summarized data for each sample as (i) count of epitopes in seroreactive regions per protein and cumulatively across all proteins for a pathogen and (ii) mean IgG enrichment value per protein calculated as sum of enrichment values divided by protein length.

Descriptive statistics were presented as frequencies and percentages, as median with interquartile ranges, or mean with standard deviation or 95% confidence interval (CI). We estimated permutation *P* values for differences between groups, permuting treatment at the cluster level (10,006 replicates). We report *P* values uncorrected and adjusted for multiple comparisons across pathogens using the Benjamini-Hochberg procedure with a 5% false discovery rate. All hypothesis tests were conducted with a two-sided significance level of *P* < 0.05.

#### Intervention group comparison

Group comparisons combined information from ages 14 and 28 months. For each pathogen, we calculated differences in the number of epitope hits and mean IgG enrichment between treatment groups using a Wilcoxon rank sum test. We compared seroprevalence between groups by estimating prevalence difference using a linear-binomial regression model, and prevalence ratio using a generalized linear model framework with a log link and a Poisson distribution to improve convergence, plus robust SEs that treated individual study clusters as independent units.

#### Incident seroconversion

We assumed that all children were at risk of seroconversion at age 14 months on the basis that the period between visit 1 (median age, 3 months) and visit 2 (median age, 14 months) coincides with a period of declining maternal immunity. That is, if a child is seropositive at 14 months of age, then it is unlikely to reflect maternal IgG. We only considered children who were seronegative at 14 months to be at risk of seroconversion at 28 months. Observations at 3 months of age (visit 1) were excluded in this analysis, and we did not anticipate seroreversion below age 27 months (*5*). Because seroconversion is an incident outcome, we estimated risk (cumulative incidence) and risk difference (RD) using a linear probability model with robust SEs at the cluster level. We estimated the ratio in seroconversion rate [or risk ratio (RR)] using a log-binomial model with robust SEs at the cluster level.

### Mathematical models to reconstruct antibody dynamics

IgG measurements at an early age such as in this study may include individuals with immunity from passive maternal transfer as well as induced by natural infection. These immunity sources are generally indistinguishable and can be separated under reasonable assumptions using catalytic models. For model fitting, we summarized the seropositives by age in months, resulting in discretized data with age range of 1 to 32 months. Given the relatively small number of samples and low seroprevalence for some pathogens, we consolidated seropositivity data from the intervention and control groups to increase the number of samples in each age class and allow for better binomial approximation of age-specific probability of infection.

For each pathogen, we fitted a modified catalytic model framework with an adaptable structure of maternal immunity to estimate (i) the force of infection defined as the rate at which susceptible individuals become infected (parameter λ) and (ii) and the rate of losing maternal immunity (parameter γ). We assumed a constant λ over time and age, which estimates long-term averages of past force of infection.

We describe population dynamics of the model with the following closed system of differential equations as demonstrated in (*28*)

dMda=−γM(a) (1)

dSda=γM(a)−λS(a) (2)

dXda=λS(a) (3)

where λ>0;γ>0.

The system solution can be expressed as

$$M\left(a\right)=e^{-\gamma a}$$ (4)

$$S\left(a\right)=\frac{\gamma }{\left(\gamma -\lambda \right)}\left(e^{-\gamma a}-e^{-\lambda a}\right)$$ (5)

$$X\left(a\right)=\frac{\gamma \left(1-e^{-\lambda a}\right)-\lambda \left(1-e^{-\gamma a}\right)}{\gamma -\lambda }$$ (6)

M(a) denotes the proportion of children of age a losing maternal immunity at a constant rate γ; S(a) denotes the proportion susceptible among children of age a born with maternal immunity; and X(a) denotes the proportion of children of age a that are seropositive from natural infection or vaccination.

For each age a, the accrued proportion of seropositive children and therefore the expected seroprevalence, π(a)=M(a)+X(a), can be calculated as 1 minus the corresponding susceptible or seronegative proportion

$$\pi \left(a\right)=1-S\left(a\right)=1-\frac{\gamma }{\left(\gamma -\lambda \right)}\left(e^{-\gamma a}-e^{-\lambda a}\right)$$ (7)

where 0<π<1.

The value π(a) could also be interpreted as probability of being seropositive at age a. The probability of infection per month unit time can be calculated as $1-e^{-\lambda }.$ Population dynamics were reconstructed with the solutions listed above and using the posterior λ and γ mean estimates. For all pathogens assessed here, we assumed all children are born with maternal immunity.

The seroprevalence data were modeled by binomial distribution with probability of success equal to π(a). The models were implemented under a Bayesian model structure using cmdStanR (v.0.5.3). The choices of priors for λ and σ (table S3) were based on prior predictive simulations of the age profile of seropositivity, which showed a wide range of possible seropositivity profiles given our choice of priors (fig. S4). The priors for λ corresponded to an average force of infection between 0.5 and 0.9, while the half Cauchy distribution priors for γ allowed for a small decay rate of maternal immunity. The priors for λ varied across pathogens but the half Cauchy prior for γ was the same across. The posterior distributions of the parameters were estimated using four chains, each with 3000 iterations and 900 burn-in. Monte Carlo Markov Chain (MCMC) convergence was assessed by visual check of chain mixing and by the Gelman-Rubin R-hat diagnostic (threshold of <1.1). The effective sample size, the estimated number of independent samples accounting for autocorrelations generated by the MCMC run, was evaluated at a threshold of >400. Parameter estimates are presented as means with 95% credible intervals (CrIs) calculated from the 2.5 and 97.5 percentiles of the posterior distributions.

## RESULTS

### Group analysis of WASH and nutrition intervention effect on immune response

We analyzed sera from 120 children randomized to the intervention (WASH + N) and control groups, 60 children in each group. There was no substantial difference in the distribution of epitope hits between the intervention and control groups (Wilcoxon rank sum test, *P* > 0.05) and in the mean seroprevalence (Table 1). Across pathogens, the mean IgG enrichment levels were lower in the intervention group versus the control group but were not significantly different between groups after adjustment for multiple comparisons (table S4). Because antibody responses are considered a measure of cumulative exposure over time, seroprevalence estimates at the median ages of 14 and 28 months were interpreted as a cumulative incidence. The overall seroconversion was highest in norovirus (98%, 96 to 100%), ETEC-ST (98%, 96 to 100%), and adenovirus (100%) (table S5). The difference in risk or cumulative incidence and the RR between groups was not significant among the pathogens assessed (*P* > 0.05) (table S6). For ETEC-ST, the children in the intervention group had a lower relative risk of seroconversion than children in the control group (RD, 3%), although not significant (table S6).

**Table 1.** IgG seroprevalence comparison of groups. Estimates are based on 60 children in each group measured twice at median ages 14 and 28 months. Ninety-five percent confidence intervals (95% CIs) and *P* values^1^ account for repeated measures within children and clusters using robust SEs. *P* values^2^ are the Benjamini-Hochberg false discovery rate–adjusted *P* values for multiple comparisons. The control group was the reference in the regression analysis. Key: C, control; I, intervention.

Pathogen	Group	*N*	# Positive	Seroprevalence (95% CI)	Prevalence difference	Prevalence ratio	*P* value^1^	*P* value^2^
ETEC-ST	C	120	116	96.7% (93.6%, 99.7%)	–	–	–	–
ETEC-ST	I	120	117	97.5% (94.9%, 100%)	0.8% (−3.2%, 4.9%)	1.0 (1.0, 1.1)	0.68	0.8
HAdV	C	120	119	99.2% (97.5%, 100%)	–	–	–	–
HAdV	I	120	120	100.0% (96.9%, 100%)	0.8% (−0.8%, 2.5%)	1.0 (1.0, 1.0)	0.32	0.66
*H. pylori*	C	120	64	53.3% (43%, 63.7%)	–	–	–	–
*H. pylori*	I	120	78	65% (56.5%, 73.5%)	11.7% (−1.7%, 25.1%)	1.2 (1, 1.5)	0.1	0.34
Measles virus	C	120	98	81.7% (75.3%, 88.0%)	–	–	–	–
Measles virus	I	120	103	85.8% (79.0%, 92.7%)	4.2% (−5.2%, 13.5%)	1.1 (0.9, 1.2)	0.38	0.66
Norovirus	C	120	117	97.5% (94.1%, 100%)	–	–	–	–
Norovirus	I	120	117	97.5% (94.9%, 100%)	0.0% (−4.3%, 4.3%)	1.0 (1.0, 1.0)	1	1
Poliovirus	C	120	74	61.7% (51.3%, 72.0%)	–	–	–	–
Poliovirus	I	120	88	73.3% (65.3%, 81.4%)	11.7% (−1.4%, 24.8%)	1.2 (1.0, 1.5)	0.09	0.34
Rotavirus	C	120	47	39.2% (30.4%, 47.9%)	–	–	–	–
Rotavirus	I	120	51	42.5% (30.2%, 54.8%)	3.3% (−11.7%, 18.4%)	1.1 (0.8, 1.6)	0.66	0.8

### Magnitude and breadth of antibody response

**Fig. 2.**
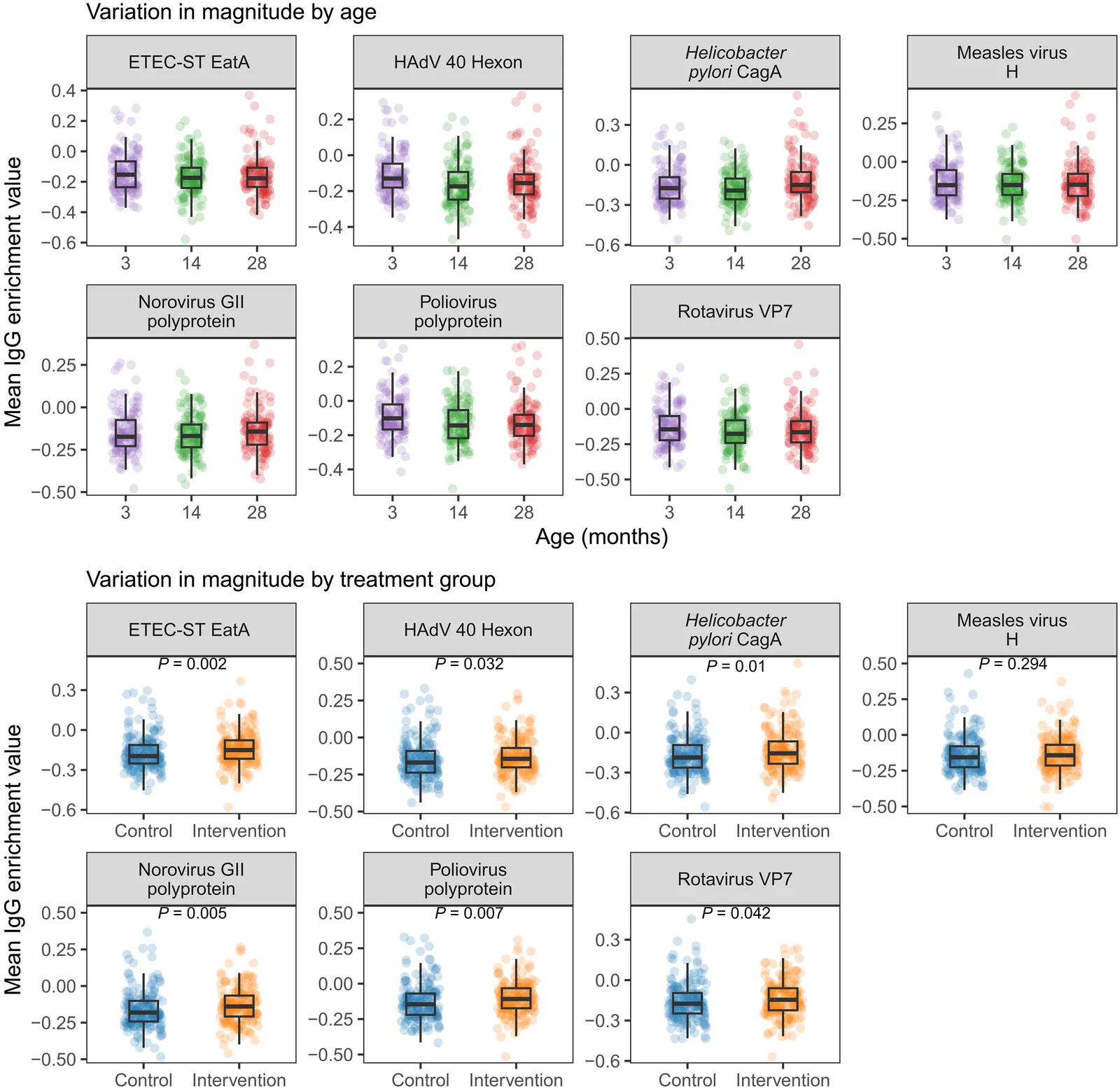
Averaged IgG enrichment scores at individual level. For each sample, the IgG enrichment scores were summarized as means by calculating the sum of normalized enrichment values per amino acid position across the protein length and then dividing by protein length (number of amino acid per protein). Along the protein length, some samples had negative normalized enrichment values, which resulted in them having a negative mean IgG enrichment value (Y axis). A positive mean value implies that the normalized enrichment values along the protein length were all positive. The *P* values in the bottom panel indicate significance level of treatment group comparison (Wilcoxon Test). The data summaries are stratified by sampling time and treatment group assigned during recruitment. Here we show data for seven example pathogen-protein pairs.

**Fig. 3.**
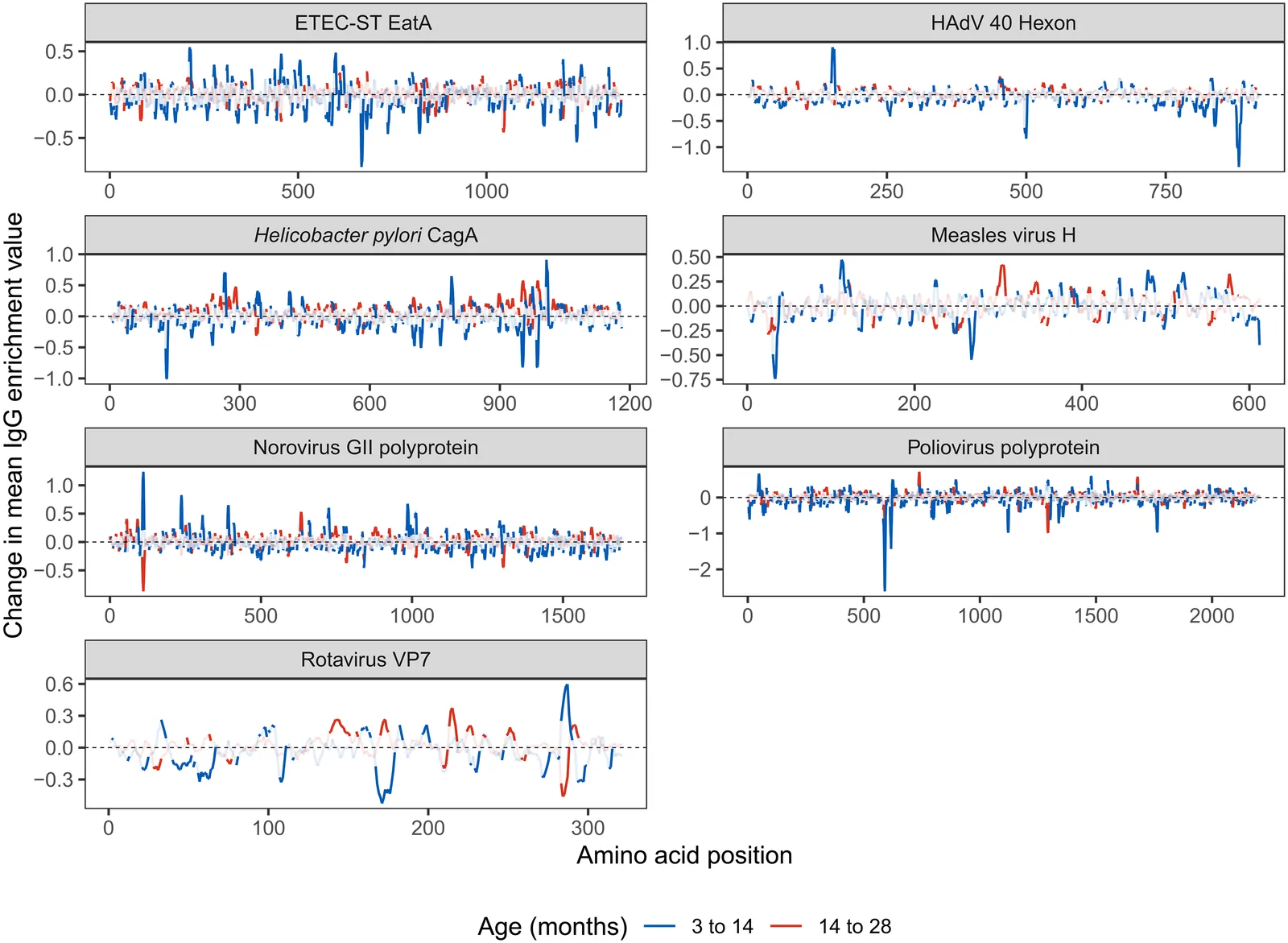
Averaged changes in IgG enrichment over time. For each amino acid position, we calculated the average enrichment value at median ages 3, 14, and 28 months and then subtracted those average values to determine the change in IgG enrichment over time. That is, [${\text{IgG}}_{14}-{\text{IgG}}_{3}$] in blue and [${\text{IgG}}_{28}-{\text{IgG}}_{14}$] in red. Values below 0 (the black dashed line) indicate a decrease in enrichment, and values above 0 indicate an increase in enrichment. Where the blue line is below 0, that indicates signatures of loss of maternal immunity at the amino acid level, where the IgG enrichment decreased between 3 and 14 months. Where the blue line is above 0, the IgG enrichment value at 14 months is bigger than the value at 3 months, which indicates gain of detectable IgG reactivity. Where the red line is above 0, that indicates signatures of immunity after infection at the amino acid level, where the IgG enrichment increased between 14 and 28 months. Where the red line is below 0, the IgG enrichment value at 28 months is smaller than the value at 14 months, consistent with a loss of detectable IgG or seroreversion. We used paired *t* test and multiple comparison adjustment methods to determine whether the changes were significantly different from zero. A significant change (Bonferroni-Hochberg adjusted *P* < 0.05) is shown as a full colored line, and the faded color indicates nonsignificant change (adjusted *P* > 0.05).

Collectively, we analyzed antibody reactivity in 360 serum samples (60 children × 3 visits per child × 2 groups) across 117 proteins in seven pathogens, ranging from 3 to 34 proteins per pathogen (table S2). We selected seven proteins for each pathogen in Table 1, which are known to be immunodominant or immunoreactive through application in serosurveillance studies or experimental studies. For the seven example proteins, the average IgG signal was similar among sampling visits but slightly higher in the intervention group (Fig. 2 and table S4) and along the protein length (figs. S5 and S6). Yet, at population level, there were varying levels of IgG signal enrichment across pathogen proteomes, and, in some protein regions, the antibody reactivity changed markedly between the sampling time points (Fig. 3). Within each age period (3 to 14 months, 14 to 28 months), there were bidirectional changes that included loss of IgG recognition in some regions of the protein concurrent with gains in recognition in other regions. We calculated, for each protein in Fig. 3, how many amino acid positions had positive (>0) and negative (<0) changes in IgG enrichment in the two time periods. Overall, there were more amino acid positions with a decrease in the average IgG enrichment value from 3 to 14 months (fig. S7). This is especially evident for longer proteins such as the viral polyproteins. From 14 to 28 months, there were more amino acid positions with an increase in the average IgG enrichment value (fig. S7). Similarly, this is evident for longer viral polyproteins and less evident for shorter proteins such as VP7. While individual-level heterogeneity in the age periods (3 to 14 months, 14 to 28 months) may not be fully captured in these observations and given the limitations of the SERA assay, it is important to note that these findings are descriptive and hypothesis generating rather than definitive evidence of specific immunologic mechanisms.

**Fig. 4.**
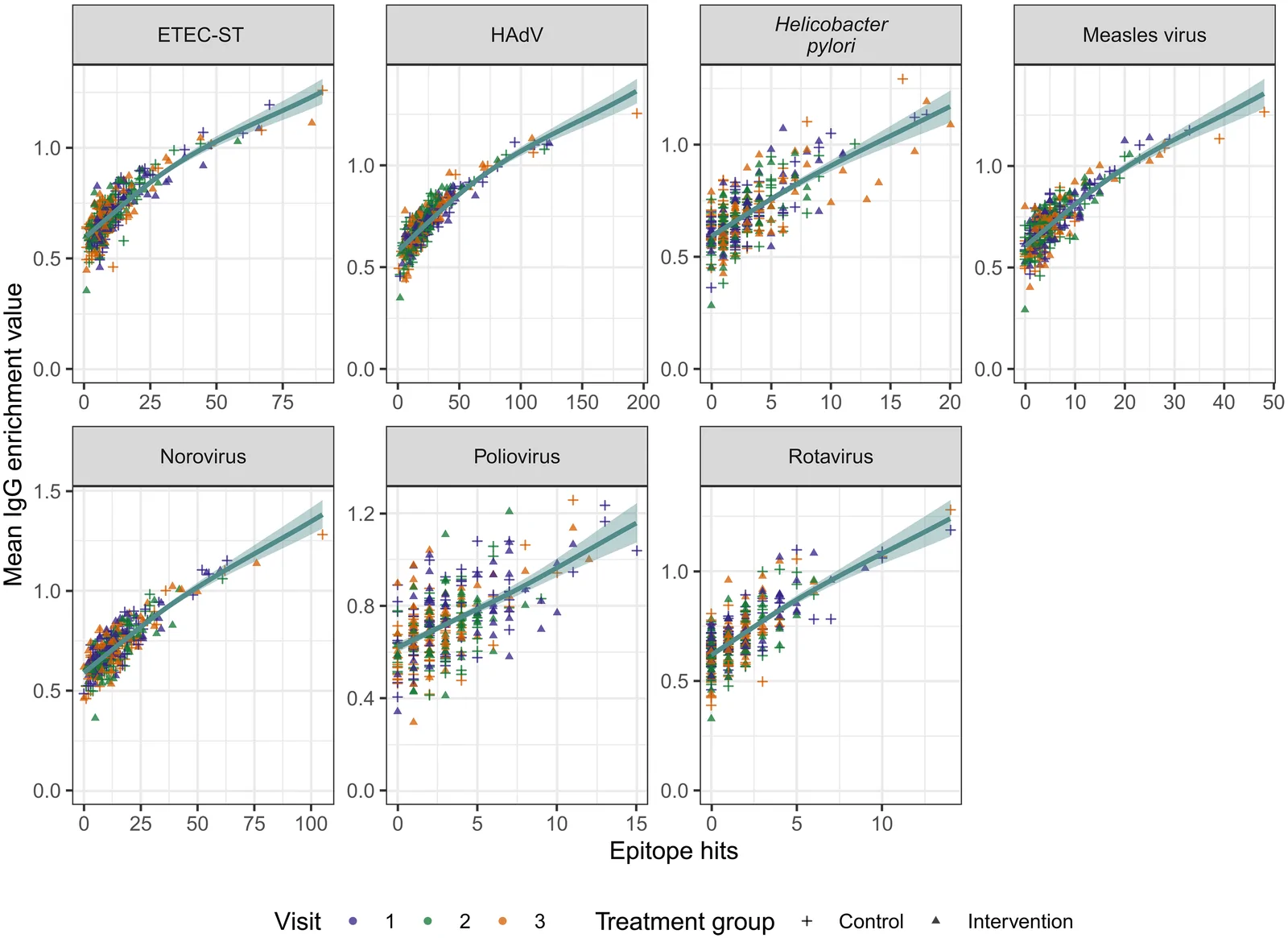
Averaged IgG enrichment versus antibody breadth. The figure illustrates association between individual-level antibody reactivity versus breadth (total count of epitope hits in a protein). For each sample, the mean IgG enrichment score was calculated across the entire protein length: Each panel has 360 data points (= 60 children per group × 2 groups × 3 sampling visits). The gray lines show a loess smoothing function, and the shading shows 95% CIs. The points are colored by sampling visit and distinguishable by treatment group.

The magnitude of IgG signal per sample increased with cumulative breadth or immune repertoire diversity measured as the total number of unique epitope hits per sample (Fig. 4). This was more prominent for pathogens with a larger number of protein components evaluated, e.g., ETEC-ST, HAdV, measles virus, and norovirus, which are, therefore, more likely to appear to have a more diverse antibody repertoire. Generally, there was minimal age effect in breadth (number of epitope hits) and magnitude (mean IgG enrichment value) (figs. S8 and S9). In the seven pathogens evaluated, there were no significant changes (adjusted *P* value of >0.05) in breadth and magnitude across age except for poliovirus, which showed a notable increase in breadth and magnitude up to 6 months of age, coinciding with the expected routine immunization window for poliovirus vaccine. At the individual level, serum reactivity and specificity were diverse across pathogen as shown in figs. S10 and S11, where amino acid residues and protein regions with enhanced seroreactivity are captured as peaks in the IgG enrichment value and by multiple overlapping linear epitope hits, respectively. We observed that the inferred seroreactive regions spanned large protein fragments or nearly full protein length (fig. S11).

### Seroprevalence

For each pathogen, we estimated seroprevalence by calculating the proportion of seropositive children at each sampling visit. Overall, seroprevalence was similar over age or time and uniform (did not differ significantly) between treatment groups (fig. S12). We also looked at the antibody reactivity of norovirus, ETEC-ST, measles virus, and poliovirus in slightly more detail. The norovirus major (VP1) and minor (VP2) capsid proteins were broadly reactive along the protein length in both genotypes (figs. S13 and S14), which suggests antigen homology, resulting in highly correlated antibody responses, although we observe a marginally attenuated seroreactivity in norovirus genotype II compared to genotype I. For genotype I, the more enhanced antibody reactivity occurred near the C terminus in both capsid proteins, and there was more seroreactivity at the central and C terminus for VP1 and at the N terminus for VP2 for genotype II. There was high serum reactivity to ETEC-ST plasmid-encoded antigens that are involved in intestinal colonization, characterized by large clusters of epitopes and seroreactive regions (figs. S15 and S16), although it did not seem to vary by intervention group or age in the six ETEC-ST antigens.

Immune responses to vaccine-targeted pathogens were also considered as a positive control: Vaccinated children should have antibodies to these pathogens. For both measles virus and poliovirus, there was no difference in the magnitude and breadth (operationalized as epitope count) of response between intervention groups, but there was higher seropositivity for the measles virus attachment glycoprotein hemagglutinin (H) compared with the fusion glycoprotein (F) (fig. S17). The intervention group was more seropositive for the measles virus F glycoprotein, whereas the control group was more seropositive for H protein (fig. S17). The overall cumulative incidence of seroconversion for measles virus and poliovirus was estimated at 82 and 67%, respectively (table S5), and the population-level model estimates showed a peak in susceptibility at 3 to 6 months of age, as discussed below.

We compared individual-level seropositivity status between the peptide array method and a multiplex bead assay and mainly focused on measles virus because the multiplex assay target for measles virus had been validated against international standards (see the Supplementary Materials) (*29*) and because viral peptides have been shown to be recognized well in immunoprecipitation assays (*30*, *31*). The two methods had moderate agreement for measles virus (62%), where the peptide array method classified 85% seropositivity and the multiplex assay classified 69% seropositivity (table S7). We estimated a sensitivity and specificity of 83 and 12%, respectively, for the SERA assay. For the other pathogens, because their antigenic targets in the multiplex bead assay had not been formally validated against international standards, we used the data to verify or rebut performance of the peptide array method by comparing seroprevalence by age (see the Supplementary Materials; fig. S18). For norovirus, the two methods consistently showed high seroprevalence and supported our decision to include norovirus in the main analysis. IgG responses measured with the multiplex bead assay showed high seroprevalence for *Cryptosporidium* spp., *Campylobacter* spp., *S. enterica* serogroup Typhi, and *Giardia* spp., broadly consistent with age-dependent patterns observed in other endemic settings (*5*), and confirmed our decision to exclude these four pathogens from the main SERA analysis.

### Model estimates of age-dependent immunity dynamics

**Fig. 5.**
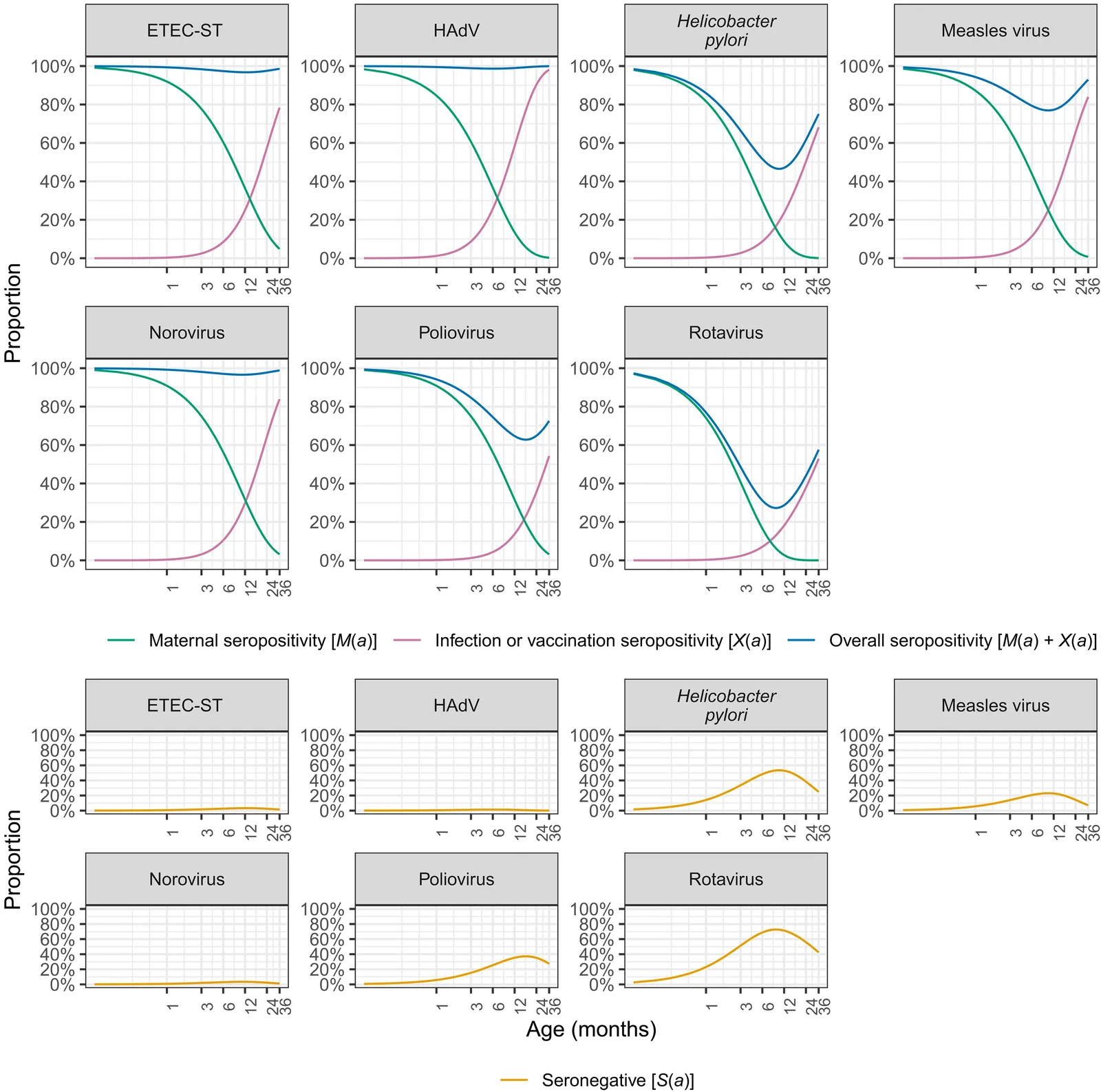
Simulated expected population immune profiles. The figure illustrates the predicted population age seroprevalence profiles based on model parameter estimates. The color of the line denotes the immunity profile: Green line represents the inferred proportion with maternal antibodies, pink represents the inferred proportion seropositive from infection or immunization (measles virus and poliovirus), blue indicates cumulative seropositivity, and orange (bottom panels) represents the proportion seronegative. Note that the age axis is log transformed for better visualization in the much younger age classes.

We implemented a modified catalytic model framework that incorporates maternal immunity to infer antibody dynamics and the expected population immune profiles. We observe substantial variation in the inferred antibody dynamics across pathogens. The inferred cumulative proportion seropositive, π(a)=M(a)+X(a) (Materials and Methods), showed the expected profile, that is, a decline then steady rise with increasing age, which was most evident for *H. pylori*, measles virus, poliovirus, and rotavirus (blue lines, Fig. 5). ETEC-ST, HAdV, and norovirus had a very high estimated cumulative seropositivity, with nearly 100% seropositivity across all study ages (1 to 36 months). We estimated a slower rate of maternal antibody decay for ETEC-ST, HAdV, and norovirus, which had ≥60% seroprevalence at 6 months of age and 40% seroprevalence at 12 months. For measles virus and rotavirus, the maternal antibody levels at the age of 6 months were estimated at 50 and 18%, respectively. At 12 months, seroprevalence due to infection and/or vaccination (for measles virus) was estimated at 20% for ETEC-ST, norovirus, rotavirus, and *H. pylori* and at 30% for HAdV and measles virus (Fig. 5).

We estimated variation in the mean duration of seropositivity due to the presence of maternal antibodies (1/γ) across pathogens, with the longest duration being 12 months [95% credible interval (CrI), 7 to 24] for ETEC-ST, followed by norovirus [10 months (95% CrI, 6 to 21)] and poliovirus [10 months (95% CrI, 7 to 15)]. The durations of seropositivity due to maternal antibodies for measles virus and rotavirus were estimated at 7 months (95% CrI, 5 to 11) and 3 months (95% CrI, 2 to 4), respectively. In all cases, the model dynamics assumed all births have maternal antibodies, that is, M(a=0)=1. This is a crude assumption and might not be applicable to all pathogens.

**Fig. 6.**
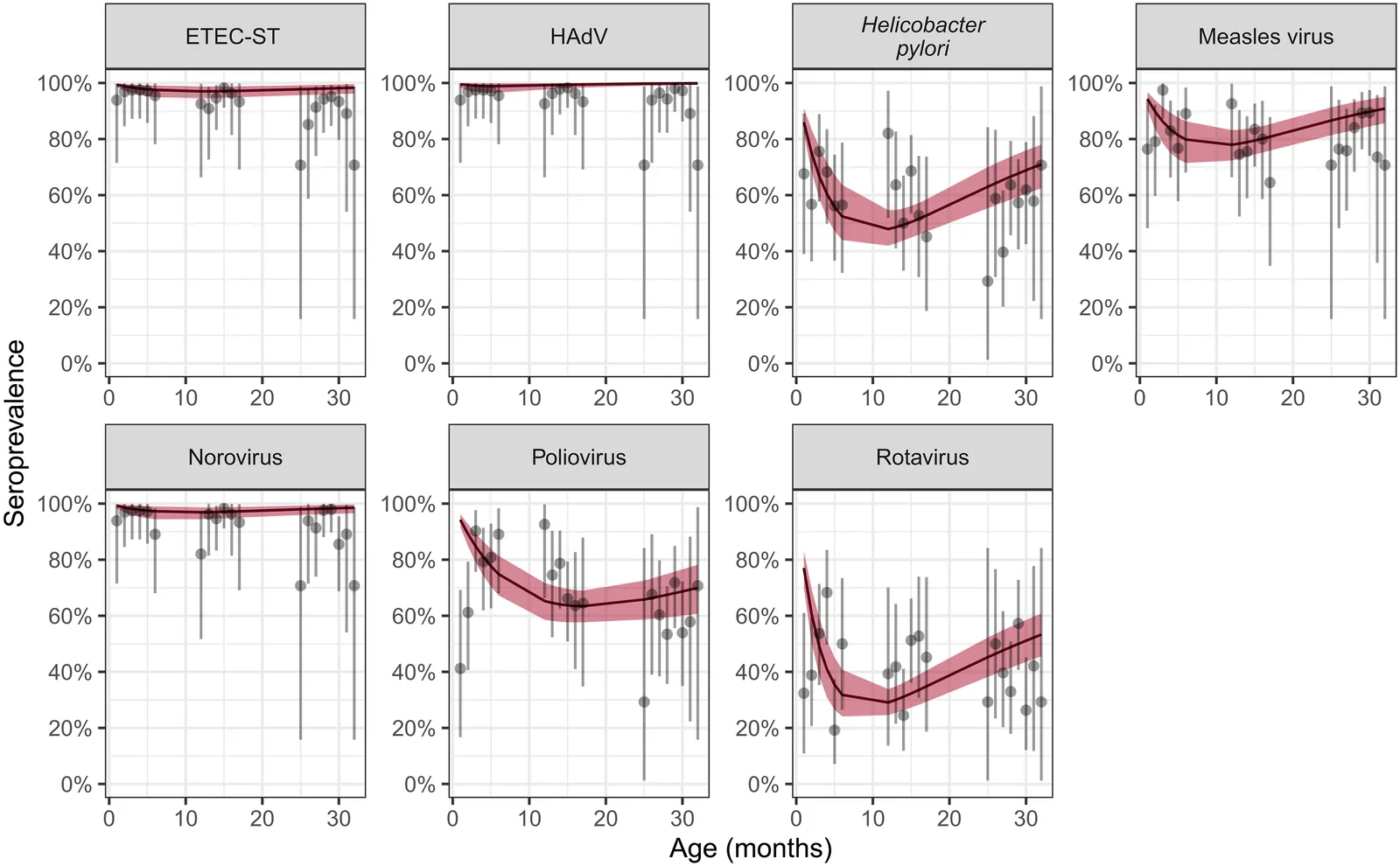
Model fitting to data. Observed (gray) and estimated (red) seroprevalence by age. The gray points and intervals indicate the point estimate and 95% binomial CIs around the observed proportion of seropositivity. The colored lines and ribbons indicate the mean and 95% CrIs of the posterior estimates of seroprevalence.

At 36 months of age, we estimated cumulative seropositivity of 95% for measles virus, 75% for *H. pylori* and poliovirus, and 60% for rotavirus. The proportion of susceptible or seronegative children, S(a), was calculated as the complement of the cumulative proportion seropositive, S(a)=1−π(a). We estimated 50 to 80% susceptibility to rotavirus in the age interval of 3 to 24 months (orange lines, Fig. 5). All parameter estimates are presented in table S8. The model exhibits a good fit to the observed data across all seven pathogens and captured the underlying pattern of decline then subsequent increase in seroprevalence (Fig. 6). The estimated seroprevalence closely matches the observed data except for the very young (ages 1 and 2 months) for poliovirus and rotavirus. We emphasize that the estimates of cumulative seropositivity and durations of seropositivity due to maternal antibodies warrant cautious interpretation given the limitations of SERA assay (see below).

## DISCUSSION

We used peptide library screening to evaluate magnitude and breadth of humoral responses to enteric pathogens in the context of public health interventions to reduce diarrhea in rural Bangladesh. Overall, we observed broad individual- and population-level antibody reactivity to pathogen proteomes characterized by varied signal intensity across the protein length as well as localized protein fragments of enriched reactivity from which we identified putative epitope variants. However, a key finding of the analysis was that the bacterial display assay had uneven performance for detecting IgG responses to known immunogenic proteins, particularly from bacterial and protozoan pathogens. We anticipate that the synthetic encoded peptides in the SERA assay lack the conformational antigenic surfaces and native modifications for optimal antibody binding specificities and may not be diagnostically useful across all pathogens. Therefore, seropositivity defined from peptide enrichment should be interpreted as an approximate or relative measure of antibody reactivity rather than a direct equivalent of gold-standard serology. High peptide reactivity does not always translate into pathogen-specific classification. In cases where no IgG signal was detected to enteric pathogens that are typically common in this setting and can be detected using ELISA or multiplex bead assays that use folded proteins (e.g., *Campylobacter* spp. and *S. enterica* serogroup Typhi), we argue that SERA assay performed poorly as a discriminatory serodiagnostic tool for these bacterial infections. Thus, while the potential breadth of testing possible through unbiased bacterial display assays is immense, in practice, they will be most valuable for detecting IgG to antigens with short linear epitopes or for discovery of linear motifs that can only be identified through comparison of known positive and negative samples (*8*).

Using a random peptide array for seropositivity assignment is challenging and imperfect. One, “crossover” signals could occur when IgG antibodies recognize linear epitopes that align with a protein or proteome of interest, but these antibodies are not (ordinarily) directed toward that pathogen. These nonspecific molecular interactions likely contributed to the some of the disparities in seropositivity estimation between the SERA assay and the multiplex bead assay (table S7 and fig. S18). Although multiplex bead assays are considered to have low measurement error (*32*, *33*), the two serological methods had a good agreement for viral antigenic targets demonstrating the use of bacterial peptide display assays to discern immunity. Two, our custom method of seropositivity calls—which did not directly account for assay sensitivity and specificity, the choice of the number of epitope hits, and the restriction of hits to seroreactive regions only—could have influenced inferences from this analysis.

Where the SERA assay estimated higher seropositivity, it could be argued that the assay was detecting more nonspecific IgG reactivity or a diverse IgG repertoire, although this should be interpreted with caution considering the limitations of serostatus assignment with the assay. The nonspecificity could also explain why IgG binding activity seemed conserved across age and the minimal differences observed between the intervention and control groups. We had hypothesized that the intervention group would have less exposure or experience fewer infections and, therefore, demonstrate lower antibody reactivity, because previous analysis of PCR detected infections in stool from the same cohort detected benefits of WASH and nutrition improvements on enteric virus and protozoan infections (table S9) (*34*, *35*). Our estimation that the intervention group had higher seropositivity for the F glycoprotein while the control group had higher seropositivity for the H glycoprotein is likely a limitation of the SERA assay and not indicative of discrepant immunoprotective and immunoregulatory responses related to the WASH and nutrition improvements (*36*).

The high serum reactivity to ETEC-ST antigens was inconsistent with the poorer performance of the SERA assay for other bacterial pathogens. This could have been nonspecific recognition to other ETEC-associated natural/native colonization factors, outer-membrane/surface antigens, and cross-reactive *E. coli* antigens. ETEC exposure can generate broad IgG responses to these factors and antigens, which may include linear-peptide binding antibodies. In this context, the assay may have detected evidence of prior immune stimulation or cross-reactive anti–*E. coli* responses.

Our bioinformatic approach for identification of antibody binding epitopes is particularly notable as we identified antigenic peptides for rotavirus reported in other studies. Antibodies to rotavirus VP7 surface glycoprotein protect against virus infectivity (*37*) and were strongly enriched in the WASH Bangladesh samples. It is important to note that these samples preceded introduction of rotavirus vaccine in Bangladesh, so IgG reflects natural infections (not vaccination). We determined potential immunodominant epitopes mostly in the central VP7 protein region, around amino acids 50 to 200 (fig. S11). This region contains antigenic site A composed of amino acids 87 to 96, which may be involved in the formation of a major protective epitope and cross-reactive neutralization (*38*, *39*). We estimated a decline in population level rotavirus-specific IgG which is marked by increasing proportion that is seronegative that peaked between 6 and 9 months of age (Fig. 5). This suggests a decline in maternal-derived rotavirus-specific IgG antibodies and is consistent immunity dynamics observed in Malawi and India (*40*, *41*). Our samples were enriched for anti–measles virus antibodies that recognized known immunodominant linear epitopes in the H protein, including amino acid residues 244 to 250, 188 to 199, and 190 to 200. The epitope at 244 to 250 is designated as “the neutralizing epitope,” and residues 188 to 199 comprise the “receptor binding epitope” (*42*). All three epitopes are targets for neutralizing measles virus infection (*42*).

Norovirus capsid proteins VP1 and VP2 are crucial antigenic components, likely to modulate immune responses (*43*). Serum samples were highly seroreactive to VP1 and VP2 as depicted by numerous predicted overlapping linear epitopes. Norovirus antibody recognition remained high between visits 1 (median age, 3 months) and 3 (median age, 28 months), suggesting persistent boosting as a function of frequent exposure. This aligns with a recent study in Nicaragua that reported new infections in early infancy, with children between 3 and 12 months of age experiencing ≥4-fold increases in norovirus IgG antibodies (*44*). We estimated waning of norovirus maternal antibodies reaching nearly 50% seroprevalence by 6 months of age, which is also consistent with seroprevalence in the Nicaraguan birth cohort (*44*).

We estimated exponential decline of seroprevalence due to maternal immunity with similar age patterns across pathogens, where seroprevalence decreases between 0 and 4 months and then increases steadily from 6 months reaching 100% by 36 months of age (Fig. 5). The estimated a median duration of maternal immunity was 10 months, and the inferred overall trends in antibody decay are consistent with the existing knowledge of duration of immunity in infants and young children (*45*). The increasing antibody seroprevalence or acquisition seemed to surpass the maternal immunity levels, which suggests that, by age 3 years, children will have attained IgG responses proportional to the level circulating in adults. Although this pattern has been observed for enteroviruses and norovirus (*44*, *46*–*48*), these findings merit cautious interpretation in light of the inherent limitations of the random peptide assays, as they may overrepresent true seroprevalence for some pathogens, especially where antibody recognition is dominated by conformational epitopes.

Our study had limitations. The samples were not evenly distributed by age resulting in small numbers at some specific ages, which could influence the precision of age-specific seroprevalence estimates. For instance, only eight children were 12 months old, only one was 25 months old, and just five were 31 months old. The primary comparisons between intervention groups were aggregated over 120 samples in each group providing reasonably good statistical power, but we refrained from estimating age-specific seroprevalence by intervention group. We also did not explore how factors such as birthweight, maternal education, nutrition, or maternal comorbidities might have affected serum reactivity, and this could be a topic for additional study. Further studies are warranted to confirm the breadth of immune response and investigate protection from infection or illness.

Our study demonstrates the use of high-throughput antibody screening to characterize antibody profiles and provides a motivation for further epitope-level serological analysis of the humoral response to enteric infections. Compared with other binding assays that have limited antigenic screening capacity and have to be preprogrammed like ELISA, bacterial peptide display assays provide high-throughput parallel serological assays are fully customizable in their antigen content and can be extended to a wide array of pathogens depending on epidemiological or clinical interest (*49*). Peptide-based serology platforms have enabled humoral immune responses profiling over a vast antigenic space, including the human virome (*50*), and helped detect and monitor pathogen-specific temporal changes in longitudinally sampled cohorts (*51*). We had hoped that the unbiased, bacterial display assay would enable broad characterization of enteric pathogen immunity across taxa, but we found that it failed to detect IgG to many pathogens of importance, a result that tempers our enthusiasm for applications beyond viral pathogens and motivates use of serological assays that include conformational proteins for many enteric pathogens of global importance.

Potential implications and public health relevance of our results include showing which pathogen classes are best captured by peptide-based serology, which is relevant for future surveillance system design. Our results reveal pathogen-specific limitations where peptide-based serology would be less predictive of true infection status and where it would provide greater surveillance value. Assay limitations notwithstanding, we show that a single laboratory platform could provide simultaneous surveillance, and, for viruses and bacteria where linear epitopes are more representative of native antigen recognition, the SERA assay provides resolution of epitope architecture not readily achieved with traditional IgG assays. Yet, for many other known immunogenic proteins to common pathogens, we failed to detect a consistent signal. A practical implication would require augmentation with alternative antigen formats (whole-protein or folded antigens) in a complementary hybrid approach. In conclusion, our results demonstrate the potential and limitations of proteome-scale antigen analyses of a wide array of pathogens of epidemiological and clinical interest.

## Supplementary Materials

### This PDF file includes:

Supplementary Methods Supplementary Results Tables S1 to S9 Figs. S1 to S18 References
