# Supplementary material for: Reconstructing pathogen-specific antibody binding epitopes and age-dependent immune signatures from proteomic-scale peptide libraries
Source: Sci Adv. 2026 Aug 19;12(34):eaec5478. doi: 10.1126/sciadv.aec5478 (PMC13488894; doi:10.1126/sciadv.aec5478)
Supplement: Supplementary file 1 [file sciadv.aec5478_sm.pdf]

Science Advances
AAAS

Supplementary Materials for

Reconstructing pathogen-specific antibody binding epitopes and age-dependent immune signatures from proteomic-scale peptide libraries

Everlyn Kamau *et al.*

Corresponding author: Everlyn Kamau, everlyn.kamau@ucsf.edu

Sci. Adv. **12**, eaec5478 (2026)
DOI: 10.1126/sciadv.aec5478

This PDF file includes:

Supplementary Methods
Supplementary Results
Tables S1 to S9
Figs. S1 to S18
References

Supplementary Methods

WASH trial in Bangladesh

Within a subsample of the cluster-randomized, controlled trial in rural Bangladesh, pregnant women were enrolled in four arms: control, water, sanitation and hygiene (WASH), child nutrition counseling plus lipid-based nutrient supplements (N), and nutrition plus WASH (WASH+N) (*52*). Among the birth cohort, biomarkers of gut inflammation (myeloperoxidase, neopterin), permeability (alpha-1-antitrypsin, lactulose, mannitol), and repair (regenerating gene 1β) at median ages 3, 14, and 28 months were measured (*53*). The objective of this subsample was to determine whether improved nutrition, WASH, or WASH+N interventions improved environmental enteric dysfunction (EED) markers (prespecified outcomes) among young children (*54*).

The substudy enrolled a subsample of clusters that was factorial and evenly balanced across the control, WASH, N, and WASH+N arms (allocation ratio 1:1:1:1). Clusters were selected based on logistical feasibility for specimen collection and transport to a central laboratory (*16*). The prespecified outcome measures were fecal EED markers (REG1B [only at age 14 months], alpha-1-antitrypsin, myeloperoxidase, and neopterin) and urinary EED markers (lactulose, mannitol, and L:M ratio) measured shortly after birth (median age, 3 months), one year after intervention initiation (median age, 14 months), and two years after intervention initiation (median age, 28 months) (*13*). Lipid-based nutrient supplements ended at age 24 months; WASH interventions ended 36 months after intervention initiation (*15*).

Multiplex bead-based serological assay

Dried blood spots had been collected on dried filter paper, which were individually sealed and stored at -20°C. A single 10 µL spot from each participant's sample was eluted overnight at 4°C in a Buffer B (1X PBS pH 7.2-7.4, 0.5% casein, 0.5% PVA, 0.8% PVP, 0.3% Tween 20, 0.02% NaN3, 3 ug/ml of E. coli extract). Eluates were further diluted with Buffer B to a final concentration of 1:400. In a custom assay, the eluted samples were incubated with a 45-plex of antigen-coupled beads to a broad set of pathogens including enteric pathogens, vaccine preventable diseases, arboviruses, and neglected tropical diseases.

Immunoglobulin G (IgG bound to beads were detected using a Luminex 200® system (Luminex Corp., Austin, TX) with xMAP® Technology. Plates included Buffer B-only wells as blanks, along with a negative control and three positive controls prepared at different concentrations from pooled sera for quality assurance. The median fluorescence intensity (MFI) with the background from the blank well subtracted out (MFI-bg) was recorded for each sample as the antigen-specific IgG. Plates were repeated if positive control monitoring targets fell outside two standard deviations of the mean across all plates. Plate controls were run in duplicate to assess the coefficient of variation (CV) within the plate and across plates. The standard deviation (SD) for each antigen's responses across plates was estimated to assess plate-to-plate variation, and a

plate was re-analyzed if more than half of the antigens deviated by >20% SD from the overall average in multiple controls. Further details of sample preparation and assay have been published (*52*, *29*).

The seropositivity cutoff for measles was determined using a standard curve using World Health Organization (WHO) standard serum (RUBI-1-94) (*29*). Seropositivity cutoffs for Giardia VSP5 and Cryptosporidium Cp17 and Cp23 were determined using receiver operating curve (ROC) analysis of known positive and negative samples. For antigens on the multiplex bead assay without external cutoffs, we used a pre-exposed cohort approach to determine seropositivity cutoffs (*53*), using the mean plus three standard deviations of samples that preceded a 100-fold increase in MFI.

Among the pathogens evaluated in this study, the multiplex bead assay included antigenic targets for (i) Measles virus – 'whole measles virus', (ii) Norovirus I and II – 'virus like particles' for genogroups GI and GII, (iii) *Salmonella* Typhi – HlyE, (iv) Giardia – VSP5, (v) Cryptosporidium – Cp17 and Cp23, (vi) Campylobacter – p18 and p39. Antigenic targets for human adenovirus (HADV), *H. Pylori*, heat-stable toxin-producing enterotoxigenic *E. coli* (ETEC-ST), Rotavirus and Poliovirus were not included in the 45-plex custom assay. Among antigenic targets that overlapped between the peptide arrays (serum epitope repertoire analysis, SERA assay) and the Luminex multiplex bead assay, only the whole measles virus on the multiplex assay has been validated against international standards (*29*). We therefore only made formal comparisons between the SERA assay and Luminex results for measles seropositivity status at the individual level.

Note, the SERA and multiplex bead assays were done through separate research projects, and this paper reports data from a subset of samples analyzed with the multiplex bead assay used to check against SERA assay results (the full multiplex serology results will be reported in a future publication).

Supplementary Results

For initial analysis, we plotted the tiling IgG enrichment values from each sample for each pathogen protein. Below is an example figure for Cryptosporidium gp60 (Cp17) protein (**Fig. S2**). In panel (A), we show a heatmap with a sample in each row and groups of samples colored by child's age. The rows are ordered by child age, in days, and the columns represent amino acid positions with each square on the heatmap indicating the IgG enrichment value. Darker green indicates higher enrichment values, and white indicates values below the protein-level 90th percentile IWAS value.

In panel (B), we show IgG enrichment values at every amino acid position for each sample tested. Each line represents an individual sample and is colored by child's age. In panel (C), we show epitopes and seroreactive regions identified in the gp60 protein. Each row represents an individual sample, and the epitopes identified in each sample are colored by child's age. See

Methods for details on calculation of IgG enrichment values using the PIWAS computational algorithm.

For each pathogen, we then summarized breadth as crude seroprevalence by calculating the proportion of children at each visit that were seropositive – i.e., had an overall count of two or more epitope hits in seroreactive regions. See **Methods** for detailed definition of *epitope hit*, *seroreactive region*, and *seropositivity*. Below is another figure showing the crude seroprevalence estimates at pathogen level, stratified by treatment arm for each sampling visit (**Fig. S3**).

These summaries were studied to exclude pathogens whose prevalence did not conform to the existing knowledge or previous evidence of epidemiology of enteropathogens in this population. From this output, we concluded that the SERA assay performed poorly in detecting conformational antigenic epitopes, and excluded the following pathogens from final analysis: (i) *Schistosoma mansoni* - schistosomiasis is not a significant burden in Bangladesh (*54*), therefore our data may have overestimated its prevalence; (ii) *Vibrio cholerae* - cholera incidence is reportedly high in Bangladesh (*55*), which challenged the low seroprevalence observed here; (iii) ETEC-LT and *Vibrio cholerae* are immunologically cross-reactive through the LTB and CTB antigens, respectively (*5*), which should have been observed if the SERA method had worked for either bacteria; (iv) Cryptosporidium spp. - cryptosporidiosis has been reported as a leading cause of morbidity and mortality among children in Bangladesh (*56*), which similarly was not reflected in our data. (v) Asymptomatic and symptomatic *Campylobacter* infections are highly prevalent among children in low- and middle-income countries, including Bangladesh (*5*, *57–59*), and therefore our method and data may have underestimated the underlying prevalence. (vi) In contrast, *Giardia* and *Strongyloides stercoralis* prevalence were deemed sufficiently low in this study population (*35*), to be measured accurately using the SERA method. (vii) *Salmonella* Typhi epitopes are likely conformational in structure (*60*) and therefore likely to have been missed using the SERA method, leading to underestimation of seroprevalence.

Additionally, IgG enrichment against *Strongyloides stercoralis* was evaluated for the SsIR protein which is not known to be immunogenic. We discuss Shigella results in a separate manuscript.

Tables

Table S1. Characteristics of children participants and timing of samples. The intervention included improved water, sanitation, and handwashing (WASH) and nutrition.

	**Control (N=60)**	**Intervention (N=60)**
**Sex**		
Female	34 (56.7%)	31 (51.7%)
Male	26 (43.3%)	29 (48.3%)
**Visit 1 – age (days)**		
Mean (SD)	122 (40.3)	97.6 (44.7)
Median [min, max]	124 [40.0, 197]	95.0 [28.0, 184]
**Visit 2 – age (days)**		
Mean (SD)	453 (36.9)	433 (35.0)
Median [min, max]	454 [370, 531]	438 [365, 529]
**Visit 3 – age (days)**		
Mean (SD)	862 (42.0)	873 (37.6)
Median [min, max]	866 [750, 947]	870 [796, 959]

Table S2. Pathogen proteome panel used in the serum epitope repertoire analysis (SERA). The number of proteins evaluated for each pathogen are shown on the last column. Several proteins sourced from different strains or isolates were used in the computational tiling algorithm, so the number of items in the 'Protein labels' column might not match the value in the 'Total proteins' column.

Pathogen	Taxa	Protein labels	Total proteins
**Enteric pathogens**			
Norovirus (GI)	Virus	ORF1, CP, VP2	6
Norovirus (GII)	Virus	ORF1, CP, VP1, VP2, POLP	9
Rotavirus	Virus	VP4, VP7, VP6	3
Human Adenovirus 40 (HAdV 40)	Virus	E1A, E1B, L4, DBP, CPH, PVIII, p100K, protease, SPIK1, SPIK2, pVI, POL, IX, PTP, IVa2, L2, pX, E4, NP, E4ORF6, 22K, E3CR1A, E3CR1B, E3RIDA, E3, UXP, E4ORF4, E4ORF3, E4ORF2, CPP, pIIIa, E3RIDB, L1	34
Human Adenovirus 41 (HAdV 41)	Virus	POL, CPP, CPH, L4, E1B, DBP, lfp, E3CR1B, E1B, IX, IVa2, leader, PTP, L1, L2pMu, UXP, E4orf2, UXP, E4orf2, pVII, L2, pVI, protease, sfp, pIIIa, E3RIDB, E3, E3RIDA, pVIII, E4orf4, E1A, E3CR1A, E4orf3, E4orf6	32
Helicobacter pylori (strain ATCC 700392/26695)	Bacteria	flgE, cagA, rplN, rplY, HefD	5
Enterotoxigenic *E. coli* with heat-stable toxin (ETEC-ST)	Bacteria	Cs1a, cfab, cs1b, cfac, cfae, cs6a, cs6b, etpa, cota, cotc, cotd, csbD, csbC, csbA, fliC, yghJ, cstA, astC, EatA	19
**Vaccine preventable pathogens**			
Poliovirus	Virus	PolyP, ORF2p	2
Measles virus	Virus	V, H, F, M, L, P, N, C	8

Table S3. Bayesian priors for λ and γ parameters.

**Pathogen**	**Parameter**	**Prior**
**ETEC-ST**	λ	$\Gamma(8{,}72)$
**ETEC-ST**	γ	$Cauchy^{+}(0.25{,}0.01)$
**HAdV**	λ	$\Gamma(8{,}72)$
**HAdV**	γ	$Cauchy^{+}(0.25{,}0.01)$
**Measles**	λ	$\Gamma(8{,}96)$
**Measles**	γ	$Cauchy^{+}(0.25{,}0.01)$
**Norovirus**	λ	$\Gamma(8{,}72)$
**Norovirus**	γ	$Cauchy^{+}(0.25{,}0.01)$
**Poliovirus**	λ	$\Gamma(4{,}72)$
**Poliovirus**	γ	$Cauchy^{+}(0.25{,}0.01)$
**Rotavirus**	λ	$\Gamma(1{,}72)$
**Rotavirus**	γ	$Cauchy^{+}(0.25{,}0.01)$
Helicobacter pylori	λ	$\Gamma(4{,}48)$
Helicobacter pylori	γ	$Cauchy^{+}(0.25{,}0.01)$

Table S4. Comparison of mean IgG enrichment between groups. The difference in the mean IgG enrichment values was estimated using a standard linear model with robust standard errors (SEs) at the cluster level. Estimates are based on 60 children in each group measured twice at median ages 14 and 28 months. 95% Confidence Intervals (CIs) and *P*-values[1] account for repeated measures within children and clusters using robust SEs. A bold font indicates $P < 0.05$. *P*-values[2] are the Benjamini-Hochberg FDR-adjusted p-values for multiple comparisons.

Pathogen	Group	N	Mean IgG enrichment value	Mean difference (95% CI)	*P* value[1]	*P* value[2]
**ETEC-ST**	Control	120	-0.147	-	-	-
**ETEC-ST**	Intervention	120	-0.113	0.0% (-0.0%, 0.1%)	0.08	0.24
**HAdV**	Control	120	-0.140	-	-	-
**HAdV**	Intervention	120	-0.105	0.0% (-0.0%, 0.1%)	0.09	0.24
**Measles virus**	Control	120	-0.133	-	-	-
**Measles virus**	Intervention	120	-0.103	0.0% (-0.0%, 0.1%)	0.19	0.28
**Norovirus**	Control	120	-0.136	-	-	-
**Norovirus**	Intervention	120	-0.105	0.0% (-0.0%, 0.1%)	0.12	0.252
**Poliovirus**	Control	120	-0.149	-	-	-
**Poliovirus**	Intervention	120	-0.104	0.0% (0.0%, 0.1%)	**0.019**	0.16
**Rotavirus**	Control	120	-0.153	-	-	-
**Rotavirus**	Intervention	120	-0.127	0.0% (-0.0%, 0.1%)	0.22	0.28
**Helicobacter pylori**	Control	120	-0.155	-	-	-
**Helicobacter pylori**	Intervention	120	-0.131	0.0% (-0.0%, 0.1%)	0.252	0.28

Table S5. Incidence seroconversion. Summary of seroconversion by pathogen calculated using a linear probability model with robust standard errors (SEs) at the cluster level.

**Seroconversion rate (SCR)**	**Pathogen**
98% (96%, 100%)	ETEC-ST
100% (100%, 100%)	HAdV
82% (76%, 88%)	Measles virus
98% (96%, 100%)	Norovirus
67% (59%, 75%)	Poliovirus
37% (30%, 45%)	Rotavirus
57% (48%, 65%)	*Helicobacter pylori*

Table S6. Incident seroconversion, comparison of groups. The difference in seroconversion per child-year (risk difference) was estimated using a linear probability model. The risk ratio in seroconversion rate was estimated using a log-binomial model. Children who had seroconverted at median age 14 months were not included in the analysis of incident seroconversions between ages 14 and 28 months. 95% confidence intervals (CIs) and *P* values account for repeated measures within children and clusters using robust standard errors. The control group was the reference in the regression analysis.

Pathogen	Group	N	No. Seroconverted	Seroconversion rate (95% CI)	Risk difference	Risk ratio	*P* value
**ETEC-ST**	Control	60	60	100.0 (100.0, 100.0)	-	-	-
**ETEC-ST**	Intervention	62	60	96.8 (92.6, 100.9)	-0.03 (-0.07, 0.01)	1.0 (0.9, 1.0)	0.13
**HAdV**	Control	60	60	100.0 (100.0, 100.0)	-	-	-
**HAdV**	Intervention	60	60	100.0 (100.0, 100.0)	-0.00 (-0.00, 0.00)	1.0 (1.0, 1.0)	0
**Measles virus**	Control	73	59	80.8 (73.6, 88.1)	-	-	-
**Measles virus**	Intervention	70	58	82.9 (73.7, 92.0)	0.02 (-0.10, 0.14)	1.0 (0.9, 1.2)	0.73
**Norovirus**	Control	61	60	98.4 (95.3, 101.4)	-	-	-
**Norovirus**	Intervention	61	60	98.4 (95.3, 101.4)	0.00 (-0.04, 0.04)	1.0 (1.0, 1.0)	1
**Poliovirus**	Control	78	49	62.8 (50.9, 74.7)	-	-	-
**Poliovirus**	Intervention	74	53	71.6 (61.4, 81.8)	0.09 (-0.07, 0.24)	1.1 (0.9, 1.4)	0.28
**Rotavirus**	Control	94	35	37.2 (27.6, 46.8)	-	-	-
**Rotavirus**	Intervention	96	36	37.5 (26.2, 48.8)	0.00 (-0.15, 0.15)	1.0 (0.7, 1.5)	0.97
**Helicobacter pylori**	Control	85	45	52.9 (40.7, 65.2)	-	-	-
**Helicobacter pylori**	Intervention	82	50	61 (50.2, 71.8)	0.08 (-0.08, 0.24)	1.2 (0.9, 1.5)	0.34

Table S7. Summary of test results of the SERA assay (peptide arrays) and Luminex multiplex bead-based assay (LMX). Test results from 358 samples measured among children at average ages 3 to 28 months, summarized by whether they were positive (+) or negative (-) by SERA and multiplex assays. Agreement is calculated based on ('true negatives[1] + true positives[4]')/total number of samples ([1+2+3+4]). The McNemar test calculates the significance in differences in proportions of the discordant data ('false negatives[2] and false positives[3]').

Assay target	[1]SERA- LMX-	[2]SERA+ LMX-	[3]SERA- LMX+	[4]SERA+ LMX+	Agreement	McNemar *P* value
Measles virus	13	97	41	207	61.5%	<.01

Table S8. Bayesian analysis of seropositivity data. Pathogen-specific posterior estimates of λ (lambda, force of infection) and γ (gamma, rate of maternal immunity waning).

Parameter	Mean	2.5th quantile	97.5th quantile	Pathogen
λ	0.07	0.03	0.12	ETEC-ST
γ	0.08	0.04	0.14	ETEC-ST
λ	0.16	0.06	0.24	HAdV
γ	0.16	0.07	0.25	HAdV
λ	0.07	0.05	0.09	Measles virus
γ	0.13	0.09	0.2	Measles virus
λ	0.08	0.04	0.14	Norovirus
γ	0.09	0.04	0.16	Norovirus
λ	0.03	0.02	0.04	Poliovirus
γ	0.1	0.06	0.13	Poliovirus
λ	0.02	0.02	0.03	Rotavirus
γ	0.3	0.22	0.4	Rotavirus
λ	0.04	0.03	0.04	*Helicobacter pylori*
γ	0.2	0.13	0.26	*Helicobacter pylori*

Table S9. Enteropathogen infections by intervention group at median age of 14 months. Results of co-occurring pathogens detected in fecal samples from children 14 ± 2 months old using quantitative polymerase chain reaction (PCR) among 120 children enrolled in the immune repertoire substudy. See (*34*), a previous publication for more details on the methods and discussion. The intervention included improved water, sanitation, handwashing, and nutrition.

Pathogen	Control (N=60)	Intervention (N=60)
**ETEC-LT**		
Negative	52 (86.7%)	45 (75.0%)
Positive	8 (13.3%)	15 (25.0%)
**ETEC-ST**		
Negative	48 (80.0%)	45 (75.0%)
Positive	12 (20.0%)	15 (25.0%)
**Shigella/EIEC**		
Negative	50 (83.3%)	52 (86.7%)
Positive	10 (16.7%)	8 (13.3%)
***Campylobacter* spp.**		
Negative	36 (60.0%)	25 (41.7%)
Positive	24 (40.0%)	35 (58.3%)
**Entamoeba**		
Negative	60 (100%)	60 (100%)
Positive	0 (0%)	0 (0%)
**Giardia**		
Negative	50 (83.3%)	53 (88.3%)
Positive	10 (16.7%)	7 (11.7%)
Cryptosporidium spp.		
Negative	54 (90.0%)	56 (93.3%)
Positive	6 (10.0%)	4 (6.7%)
**Salmonella**		
Negative	60 (100%)	60 (100%)
Positive	0 (0%)	0 (0%)
**Schistosoma**		
Negative	60 (100%)	60 (100%)
Positive	0 (0%)	0 (0%)
H. pylori		
Negative	58 (96.7%)	58 (96.7%)
Positive	2 (3.3%)	2 (3.3%)
**Norovirus GI**		
Negative	55 (91.7%)	57 (95.0%)
Positive	5 (8.3%)	3 (5.0%)
**Norovirus GII**		
Negative	48 (80.0%)	50 (83.3%)
Positive	12 (20.0%)	10 (16.7%)
**Adenovirus 40/41**		
Negative	55 (91.7%)	56 (93.3%)
Positive	5 (8.3%)	4 (6.7%)
**Rotavirus**		
Negative	60 (100%)	59 (98.3%)
Positive	0 (0%)	1 (1.7%)

**Strongyloides**<br>Negative<br>Positive	 60 (100%)<br>0 (0%)	 60 (100%)<br>0 (0%)
V. cholerae<br>Negative<br>Positive	 59 (98.3%)<br>1 (1.7%)	 60 (100%)<br>0 (0%)

Figures

Fig. S1. Participants enrollment workflow.

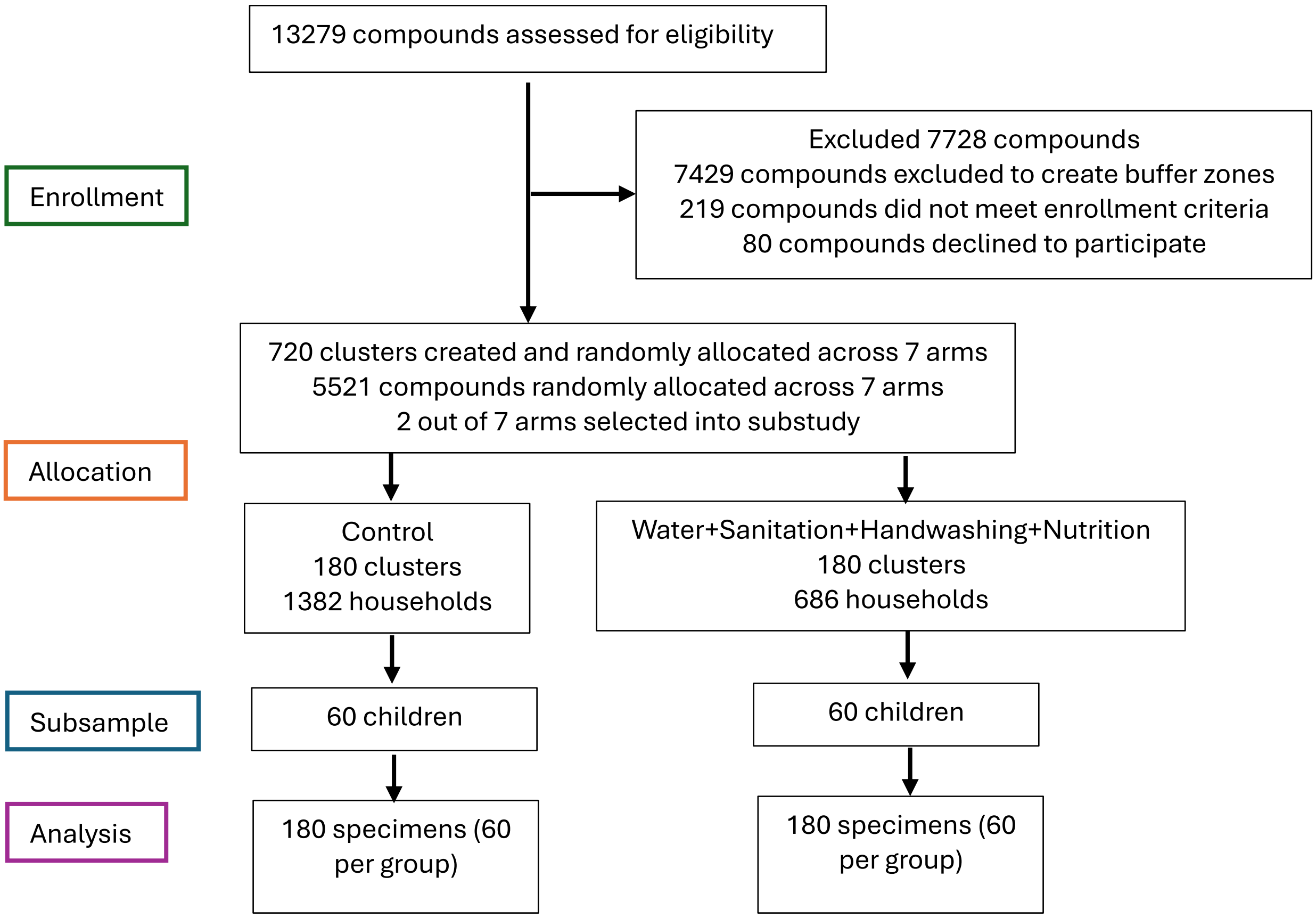

Fig. S2. Tiling IgG enrichment values across samples. Cryptosporidium Cp17 protein IgG enrichment and epitope hits.

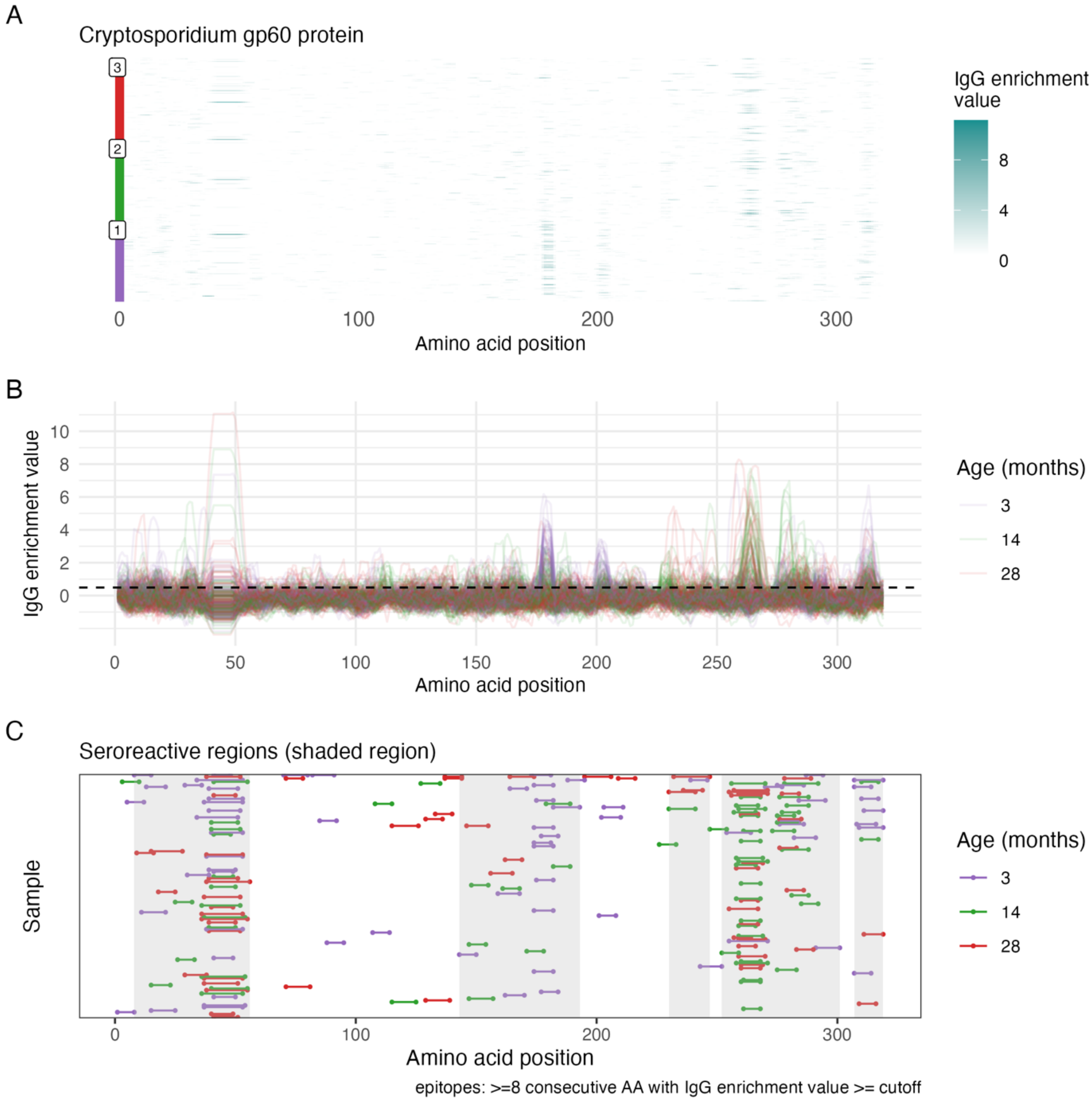

Fig. S3. Estimated seroprevalence. IgG seroprevalence for all pathogens analyzed using the bacterial peptide arrays.

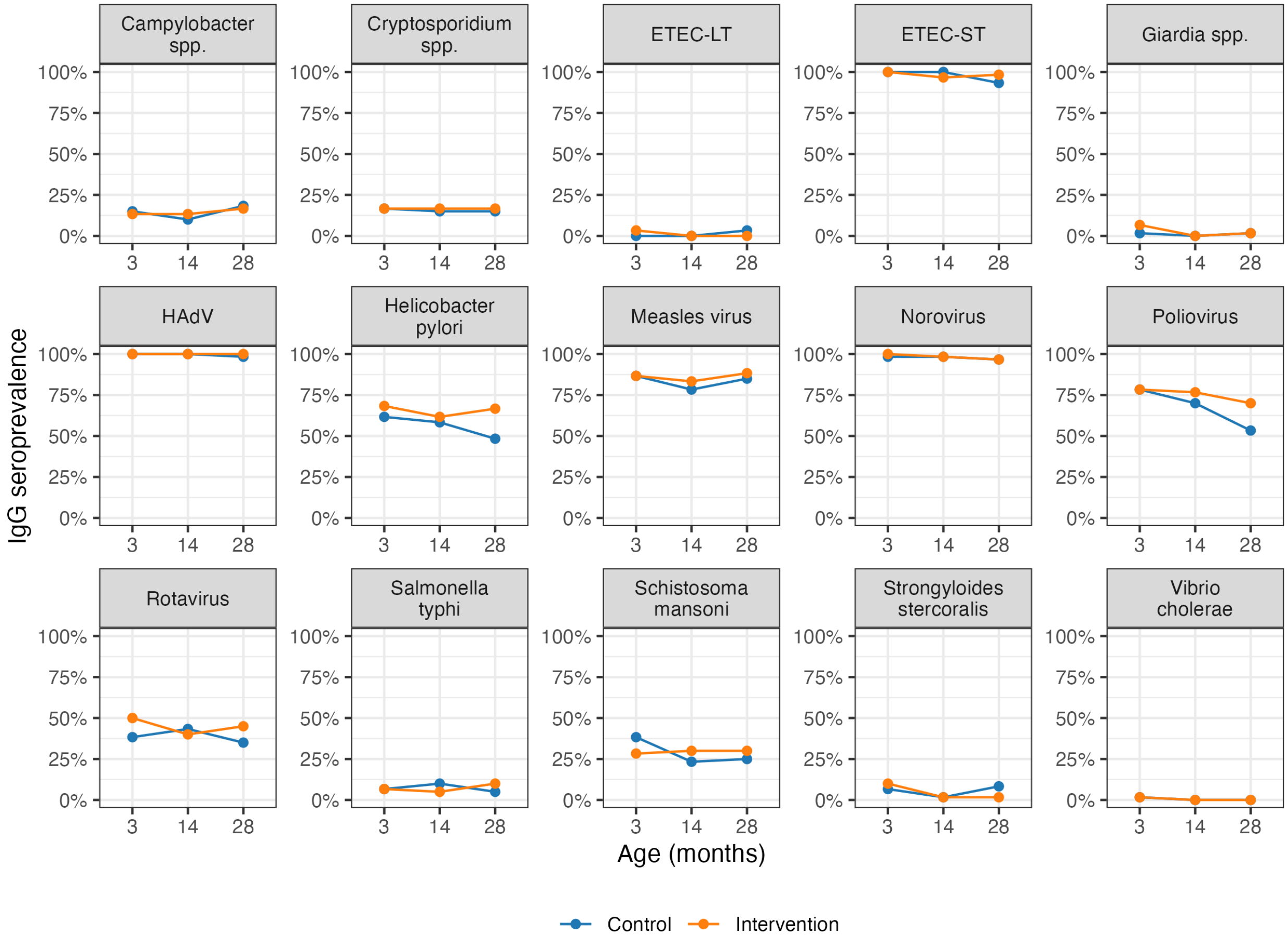

Fig. S4. Prior predictive simulations. Prior predictive simulations of the age-profile of seropositivity to assess the appropriateness of priors of model parameters λ and γ, i.e., the implications of priors in terms of what they say the data will look like. The shaded area is the 95% credible interval, and the solid line is the mean estimate of seropositivity. The notation $\Gamma(\alpha, \beta)$ refers to Gamma distribution with shape α and rate β, while $\mathrm{HC}(\mu, \sigma)$ denotes half Cauchy distribution with mean μ and standard deviance σ.

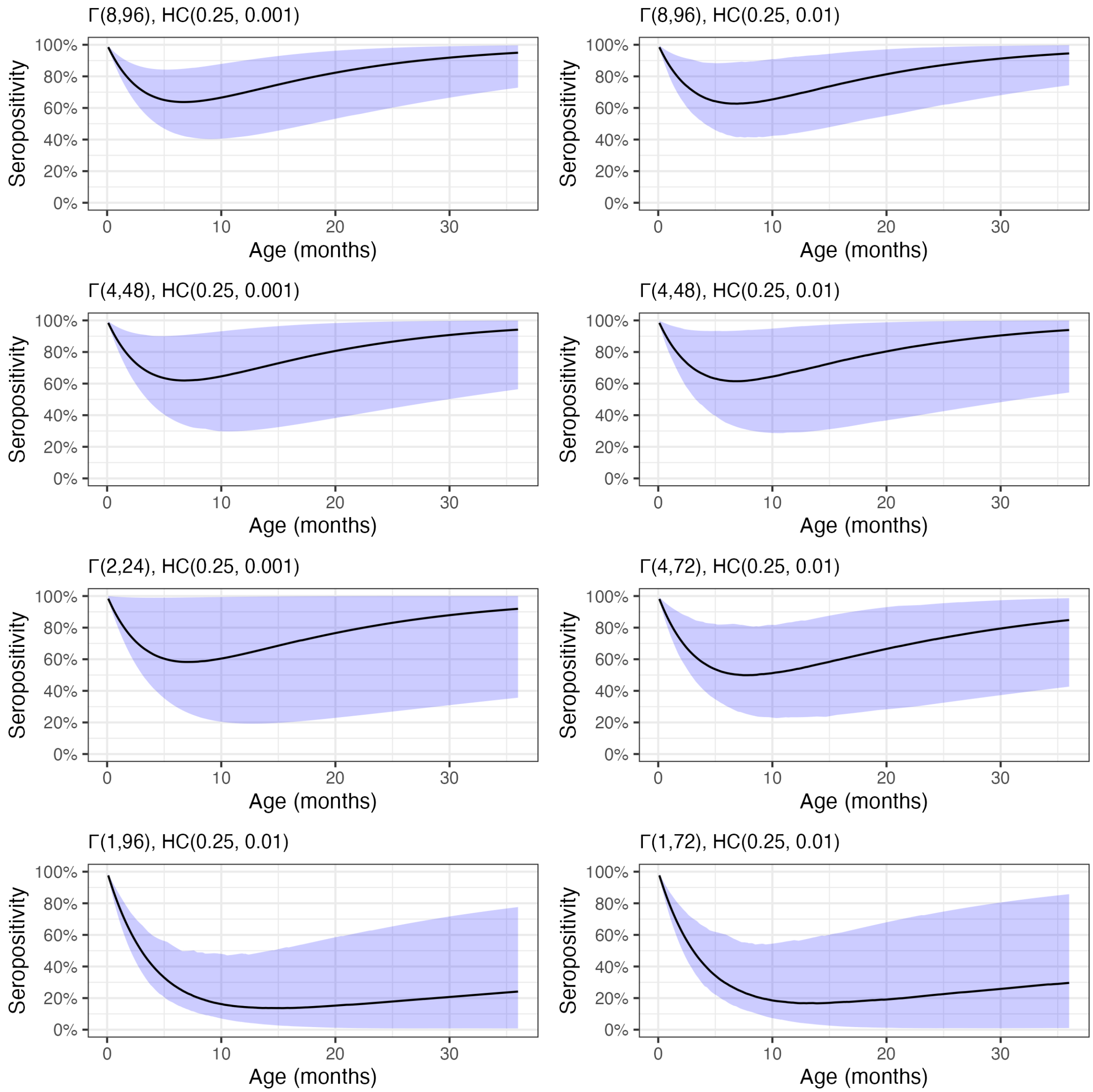

Fig. S5. Averaged enrichment scores per amino acid (AA) position. Line profiles of antibody binding for seven pathogen-protein pairs. At each AA position, the mean IgG enrichment values were calculated by the summing enrichment values at each age and dividing by number of samples (n=120 per sampling timepoint). The averaged IgG enrichment values are colored by age.

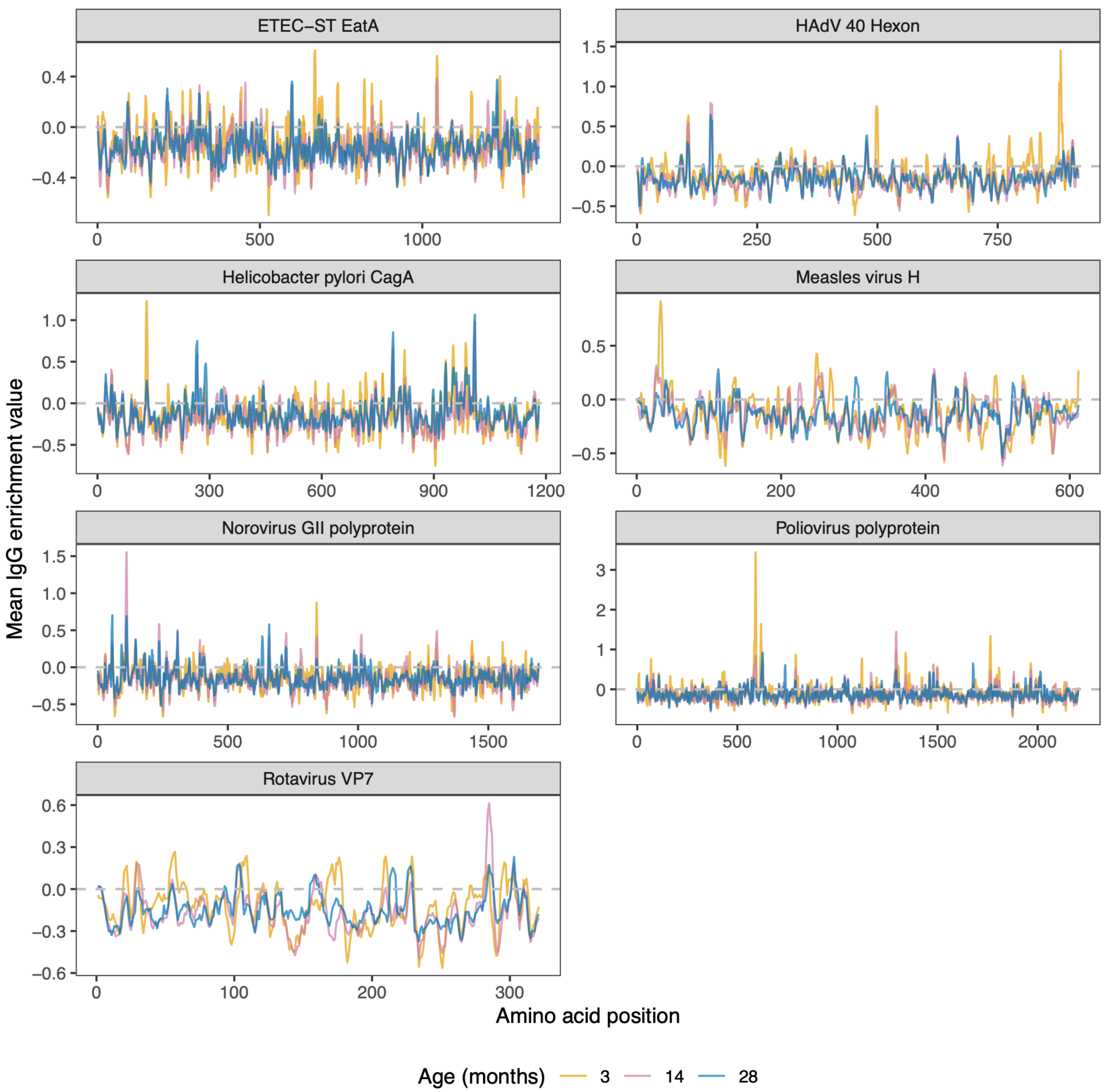

Fig. S6. Averaged enrichment scores per amino acid (AA) position. Line profiles of antibody binding by treatment group for seven pathogen-protein pairs. As in Fig. S5, we calculated the average IgG enrichment score by treatment group at each amino acid position.

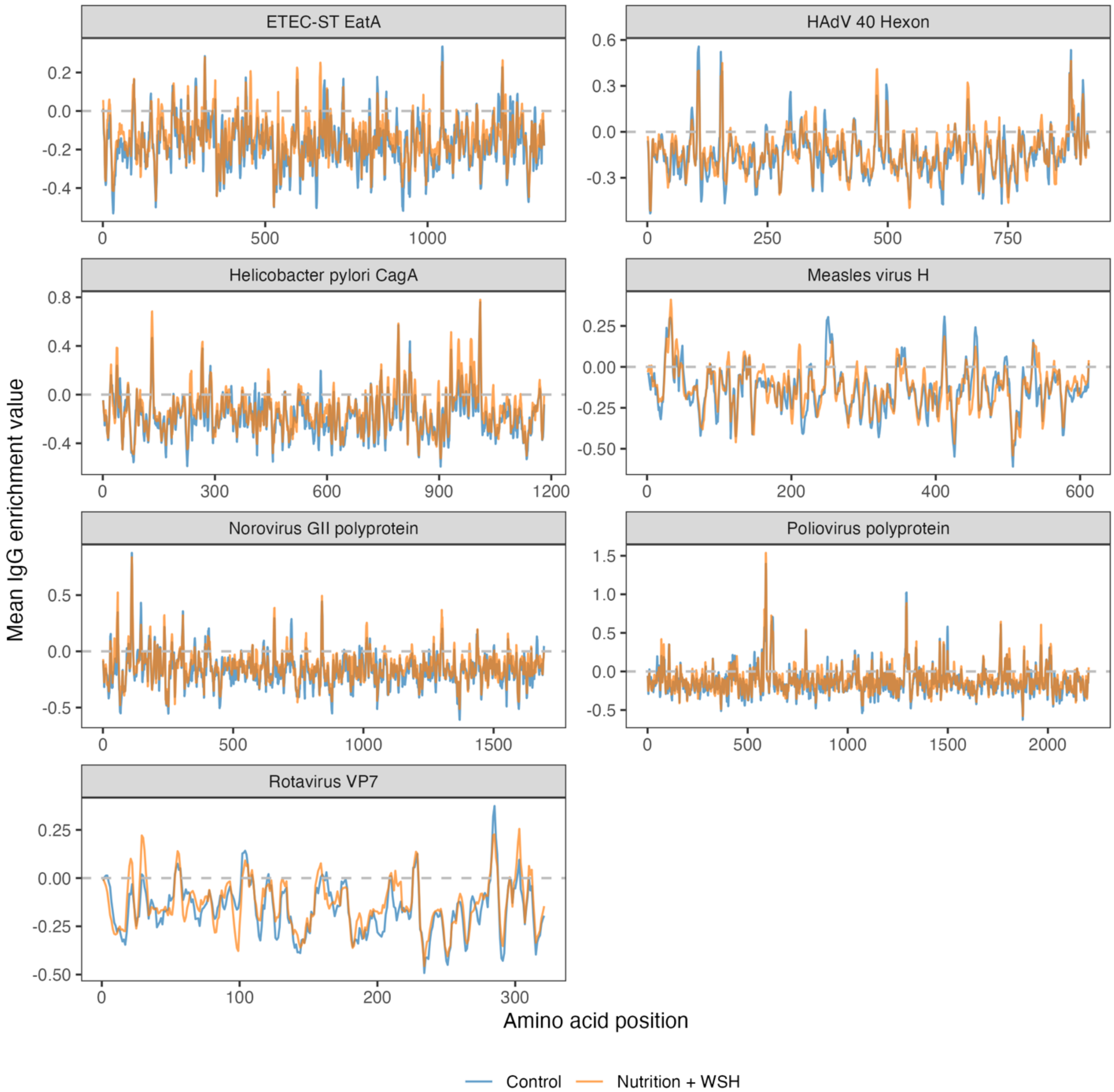

Fig. S7. Graphical summary of protein-level bidirectional changes in mean IgG enrichment values. This figure shows the number of amino acid positions experiencing changes in the mean IgG enrichment value at the two periods, age 3 to 14 months versus 14 to 28 months, as shown in Fig. 3.

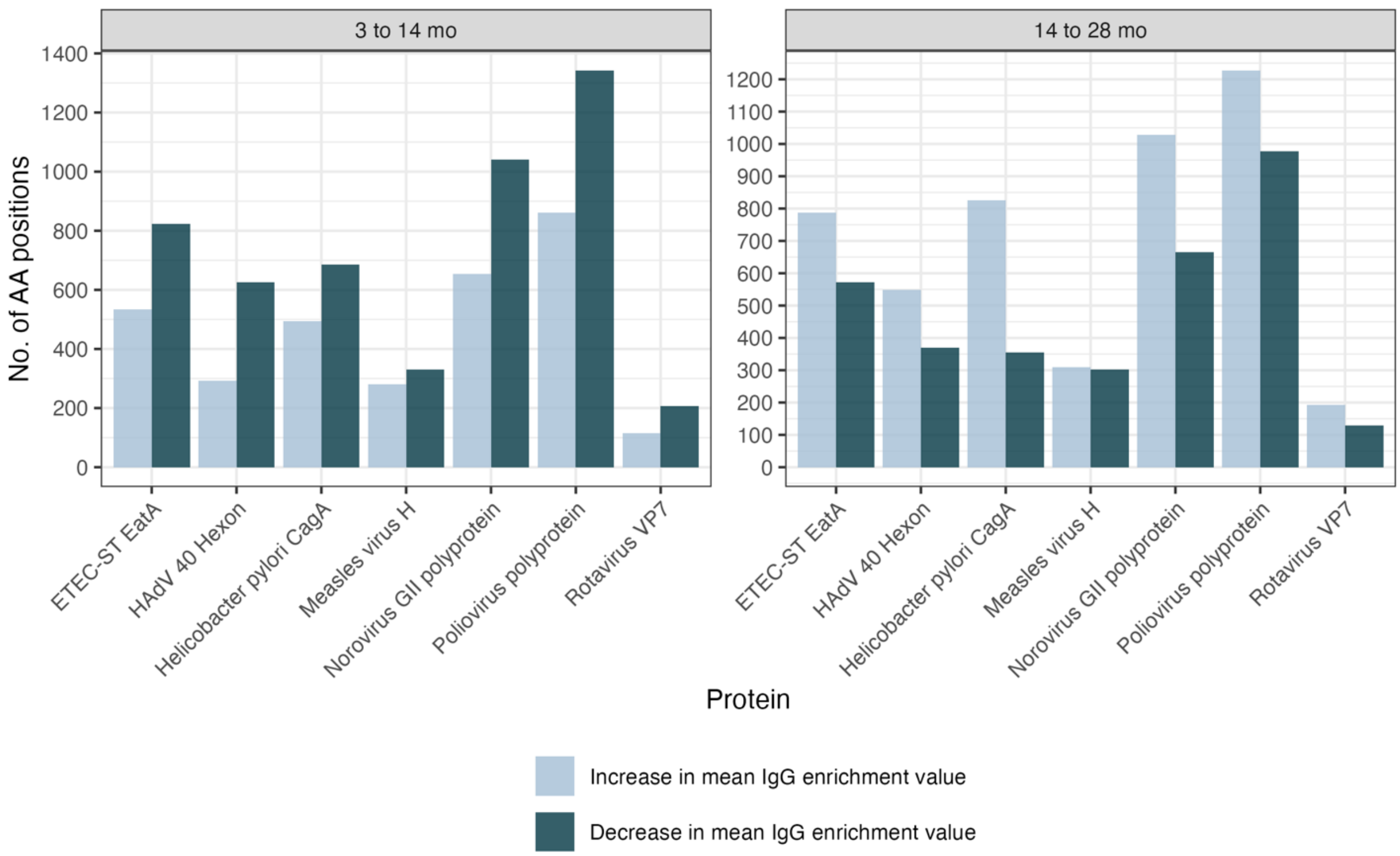

Fig. S8. Breadth. Distribution of epitope hits by child age in months. Each dot represents a single sample, where all the epitopes across proteins of a pathogen have been totaled into a single quantity. The continuous lines depict fitted smooth functions using a generalized additive mixed model with a random effect structure for clusters. Shaded areas indicate model estimated 95% confidence intervals.

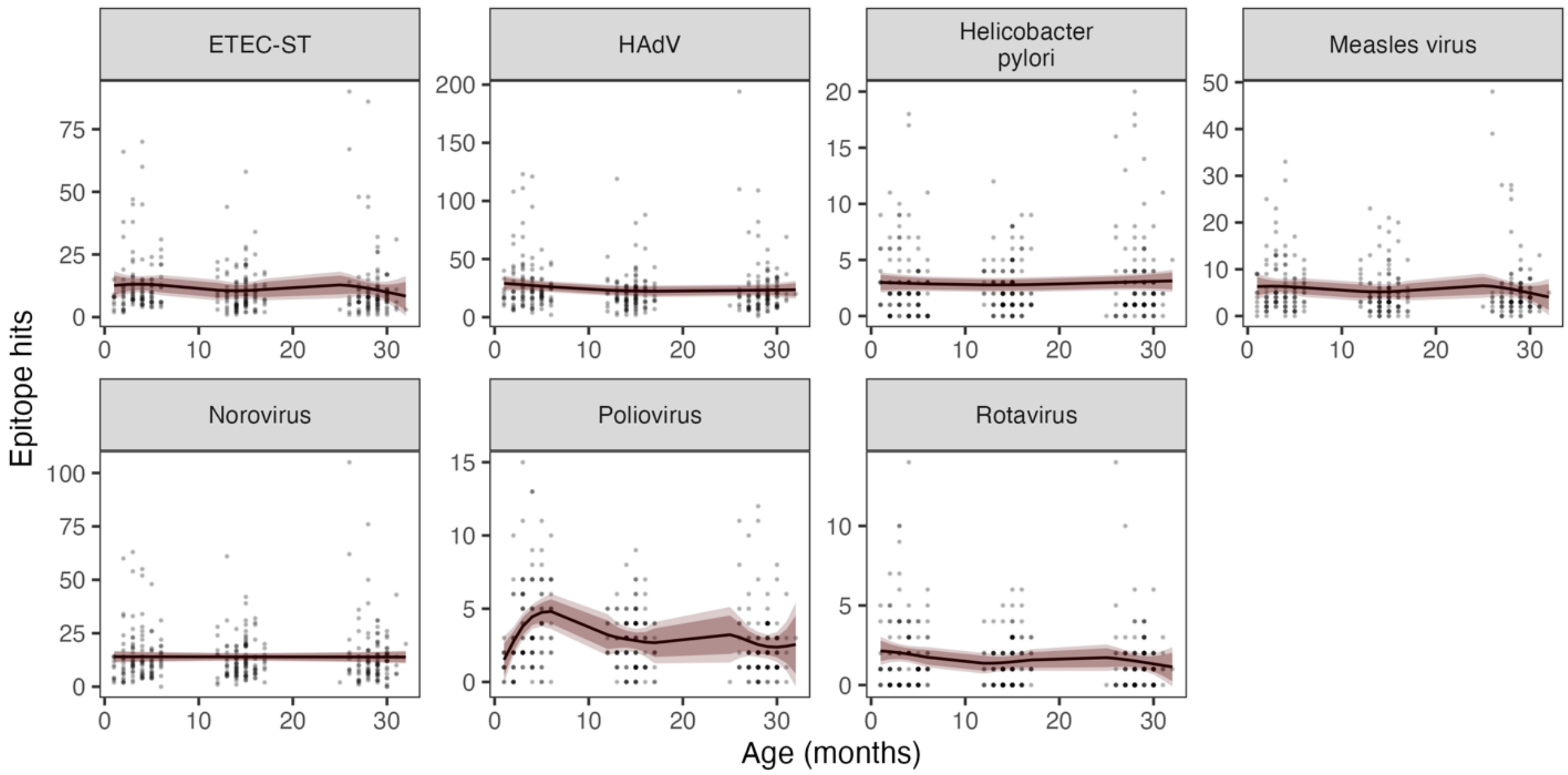

Fig. S9. Magnitude. Distribution of averaged IgG enrichment values by child age in months. Each dot represents a single sample, where the mean IgG value is calculated across all proteins in a pathogen. The continuous lines depict fitted smooth functions using a generalized additive mixed model with a random effect structure for clusters. Shaded areas indicate model estimated 95% confidence intervals.

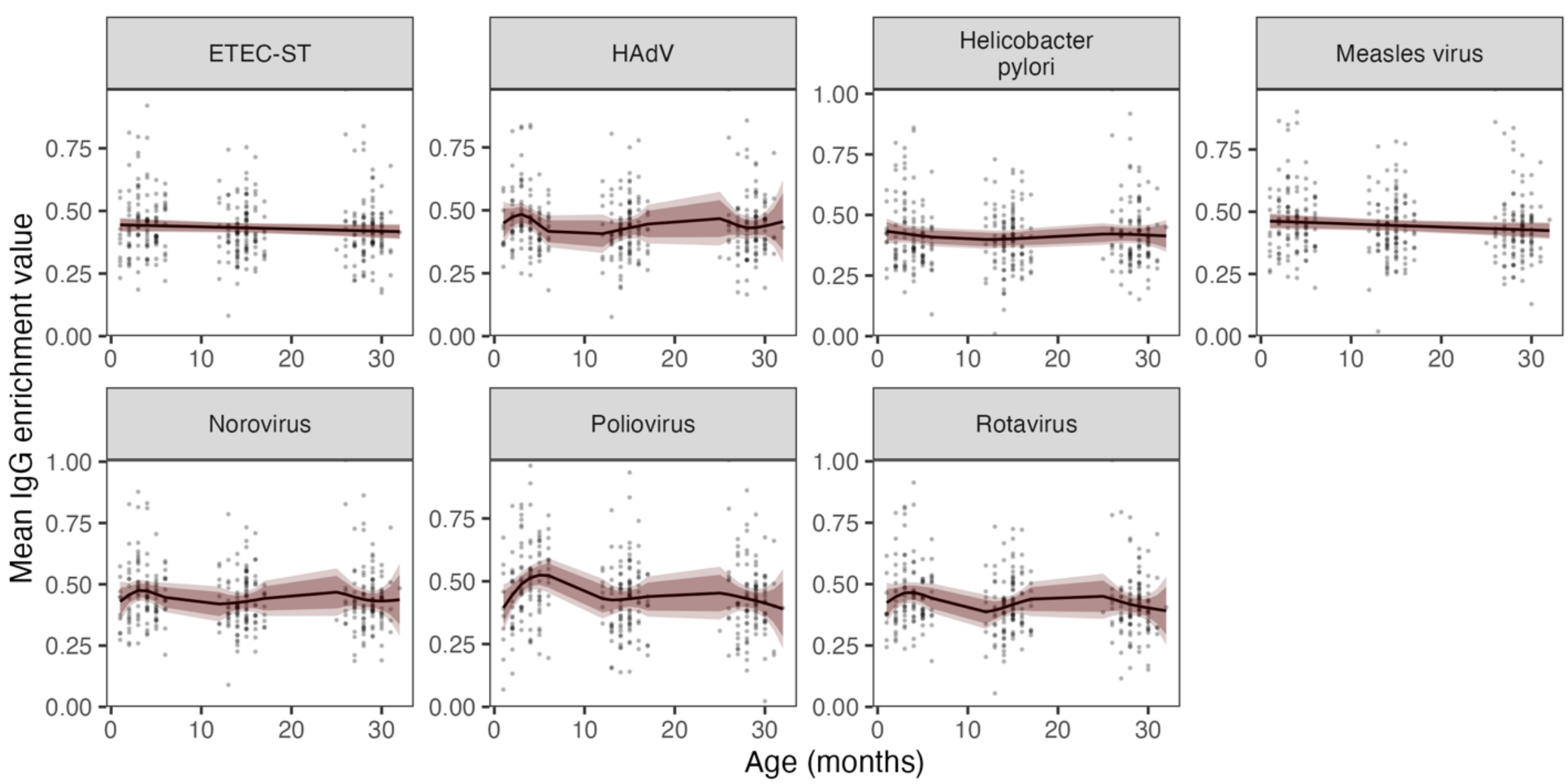

Fig. S10. Individual-level IgG seroreactivity. Line profiles of each sample showing the normalized IgG enrichment value per amino acid position for seven pathogen-protein pairs. Each line is colored by median sampling age.

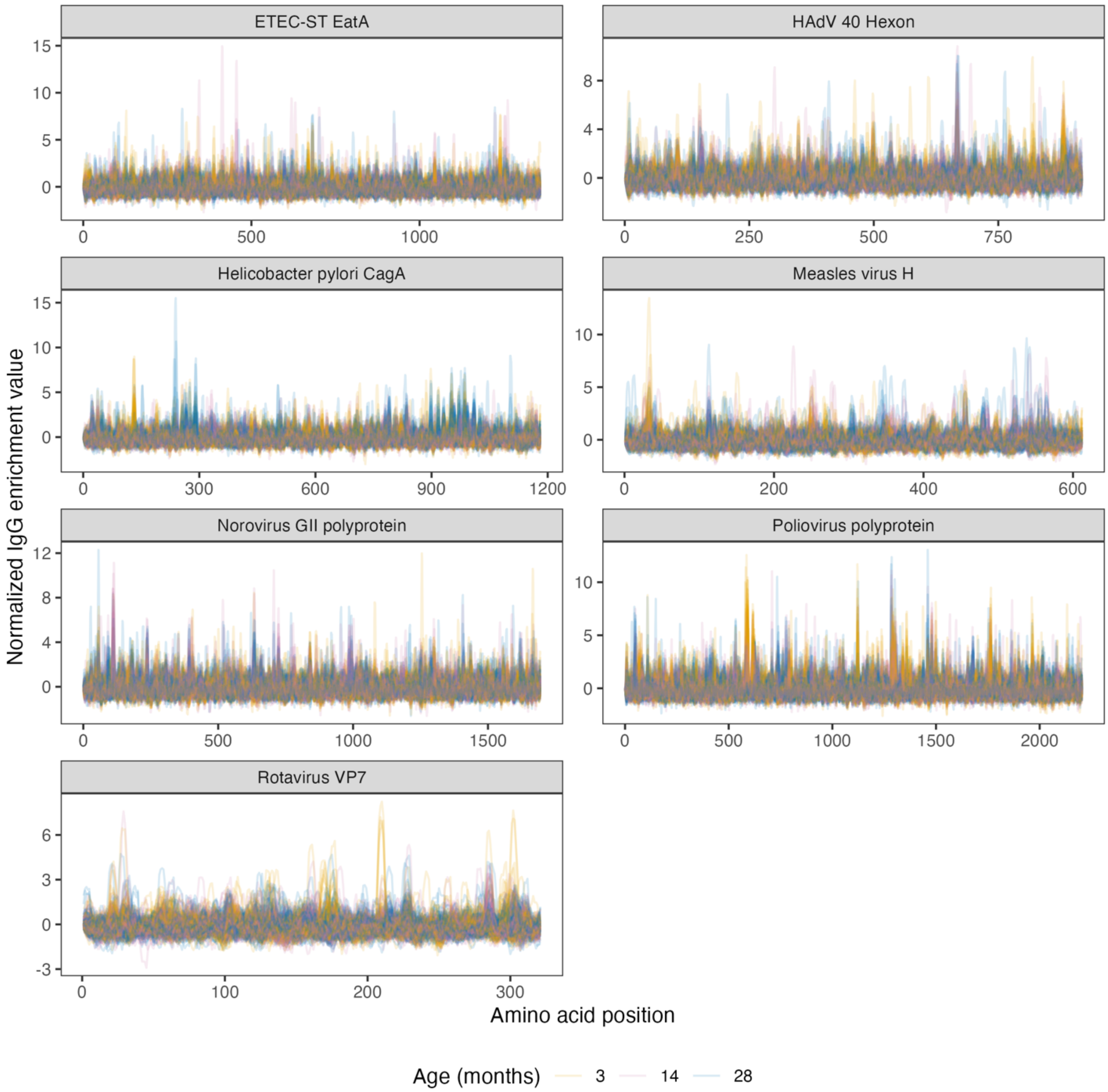

Fig. S11. Individual-level IgG seroreactivity. Epitope hits for selected pathogen-protein pairs. For each protein, epitopes were determined as a stretch of eight or more amino acids with an IgG enrichment score greater or equal to the overall protein-level 90th percentile enrichment score. Each row contains individual samples. Rows are ordered by breadth (the number of epitopes in a sample). The grey shading indicates a seroreactive region determined as a highly reactive protein fragment containing five or more overlapping epitopes in each residue. Epitopes are colored by median age at sampling.

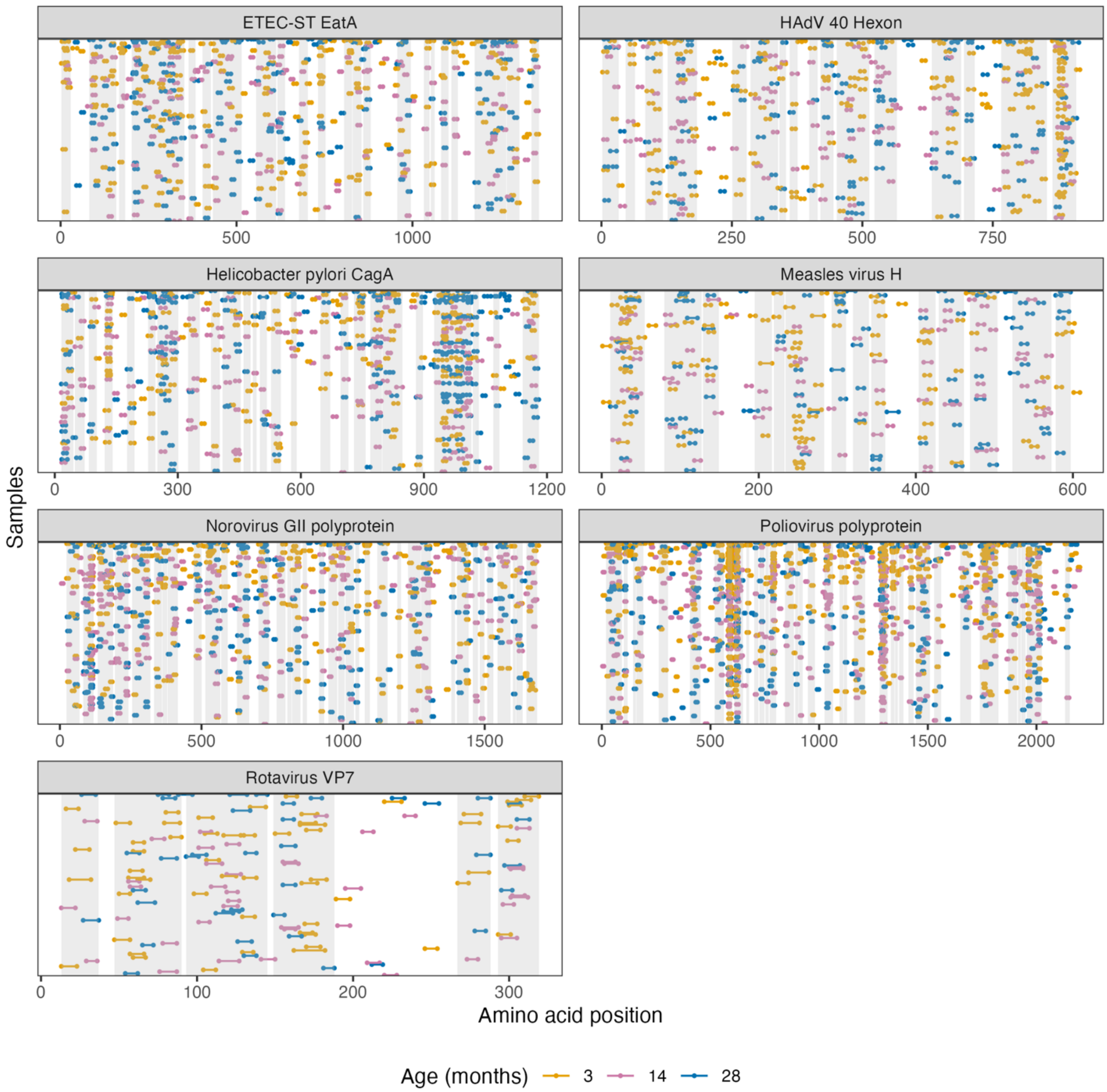

Fig. S12. IgG seroprevalence. Pathogen-specific seroprevalence determined as the proportion of children with two or more epitope hits in seroreactive regions across a pathogen's proteome. The data is stratified by treatment group at each median age. The mean IgG seroprevalence and 95% bootstrap confidence interval (1000 bootstrap replicates) are shown.

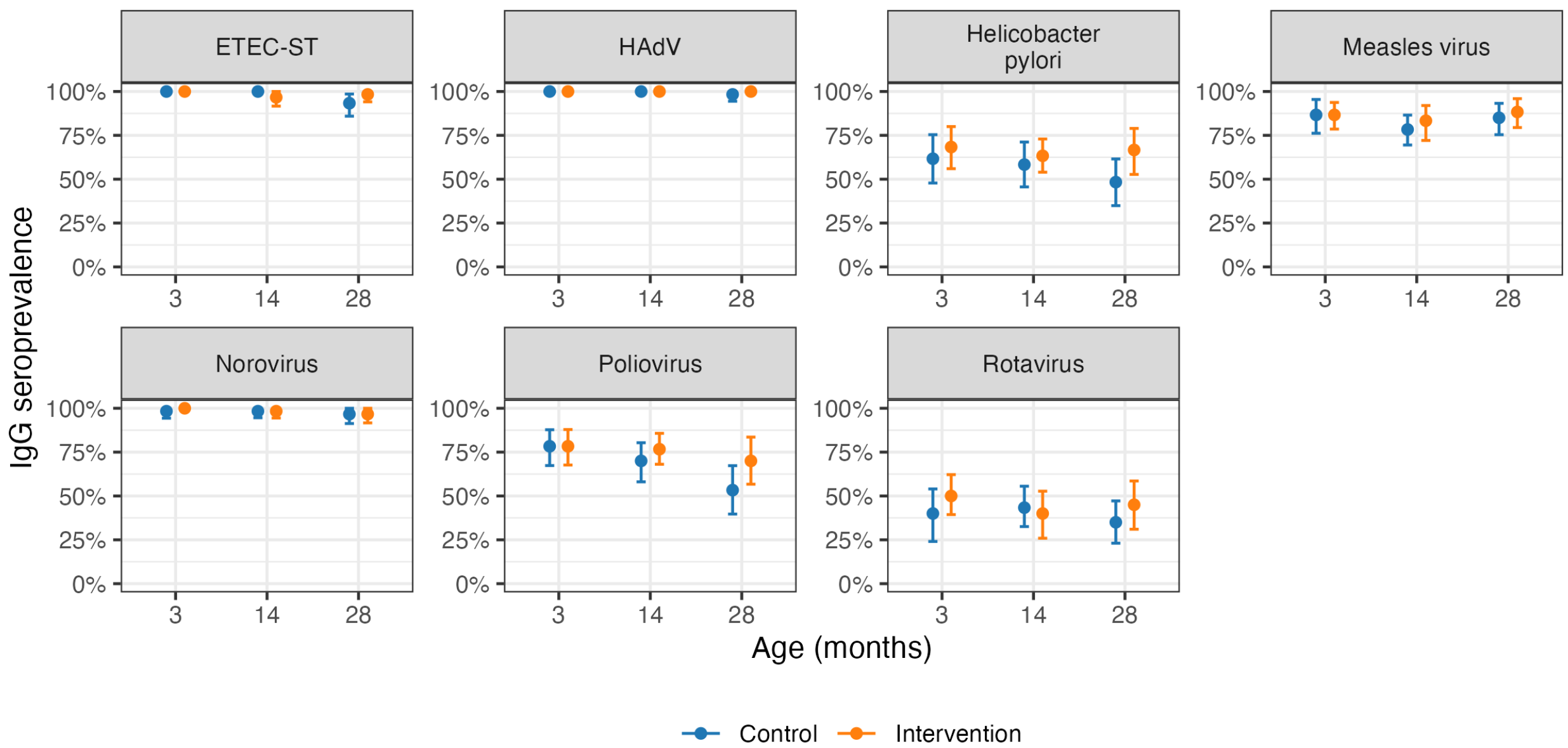

Fig. S13. Norovirus changes in antibody reactivity by age. Line profiles showing changes in IgG reactivity. For each amino acid (AA) position we calculated the average enrichment score by age and then subtracted the mean values to determine the change in IgG enrichment. That is, $[IgG_{14} - IgG_3]$ and $[IgG_{28} - IgG_{14}]$. Values below 0 (black dashed line) indicates change in the negative direction and above 0 the change is in positive direction – (i) if the signal at age 3 is larger than at age 14, it shows as a blue line below 0, if smaller, it shows as blue line above 0; (ii) if the signal at age 14 is larger than at age 28 it shows as a red line below 0, if smaller, it shows as a red line above 0. This figure also showcases inferred seroreactivity in different Norovirus genotypes and strains – the middle and bottom panels compare VP1 and VP2 proteins in two genotype GII strains, marked (1) and (2).

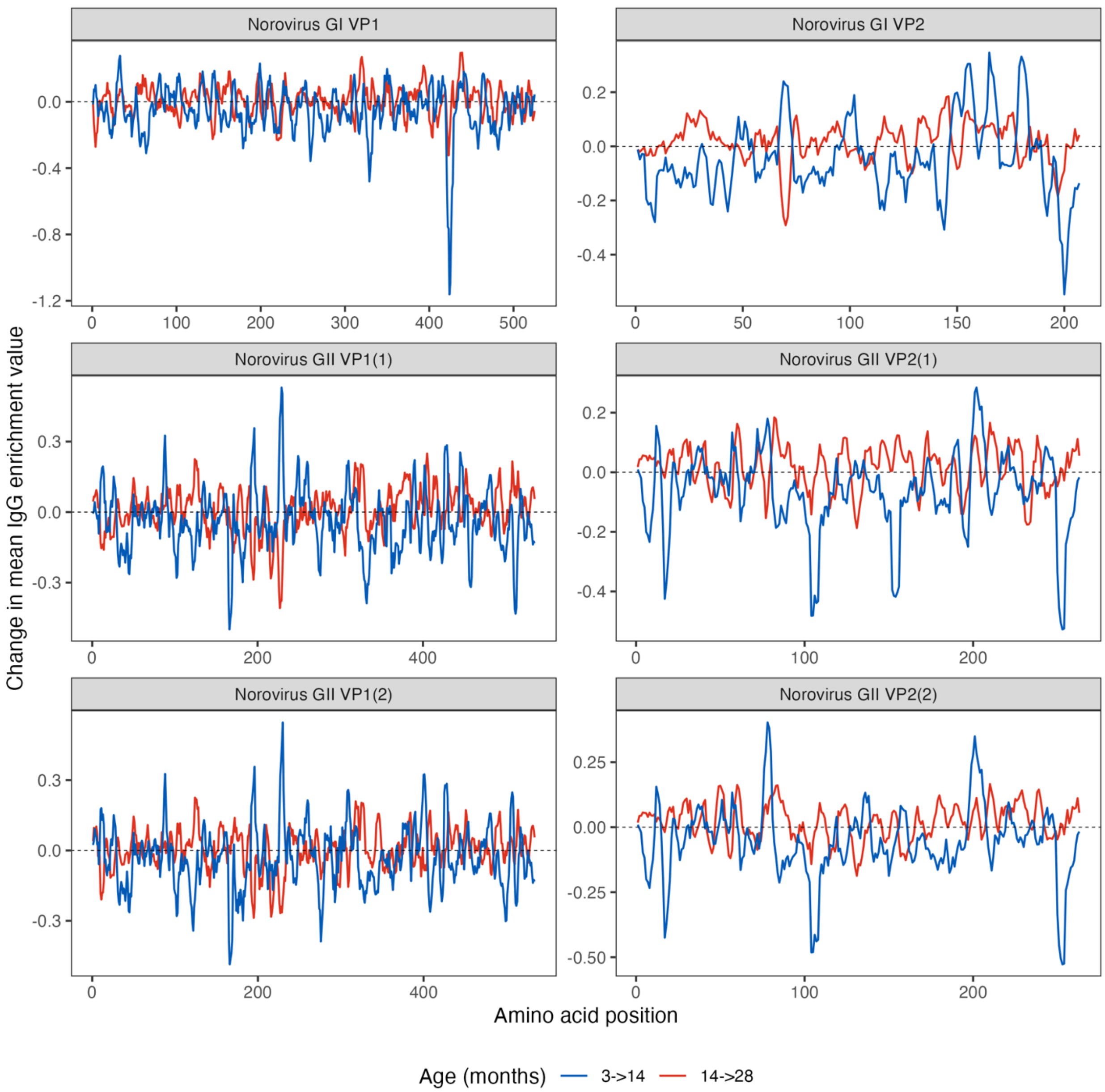

Fig. S14. Norovirus magnitude by age and treatment group. Averaged IgG enrichment scores at individual level for Norovirus VP1 and VP2 proteins. For each sample, the enrichment scores were summarized as means by calculating the sum of enrichment values across the protein length and dividing by protein length (number of amino acids per protein). The data summaries are stratified by age and treatment group. This figure also showcases inferred seroreactivity in different Norovirus genotypes and strains – the middle and bottom panels compare VP1 and VP2 proteins in two genotype GII strains, marked (1) and (2).

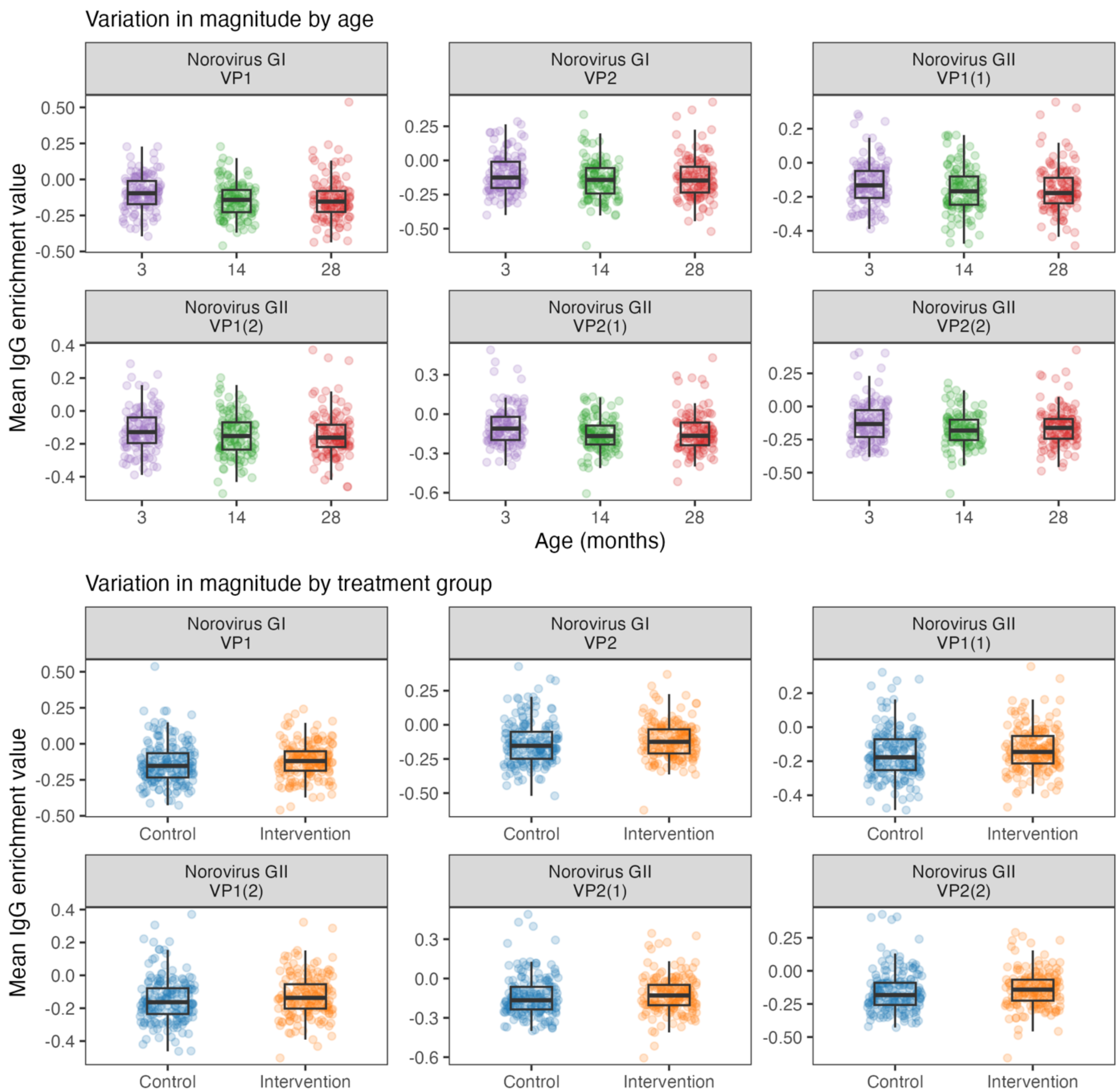

Fig. S15. ETEC-ST – line profiles of changes in antibody reactivity by age for a select number of proteins. For each amino acid (AA) position we calculated the average enrichment score by age and then subtracted the mean values to determine the change in IgG enrichment. That is, $[IgG_{14} - IgG_{3}]$ and $[IgG_{28} - IgG_{14}]$. Values below 0 (black dashed line) indicates change in the negative direction and above 0 the change is in positive direction – (i) if the signal at age 3 is larger than at age 14, it shows as a blue line below 0, if smaller, it shows as blue line above 0; (ii) if the signal at age 14 is larger than at age 28 it shows as a red line below 0, if smaller, it shows as a red line above 0.

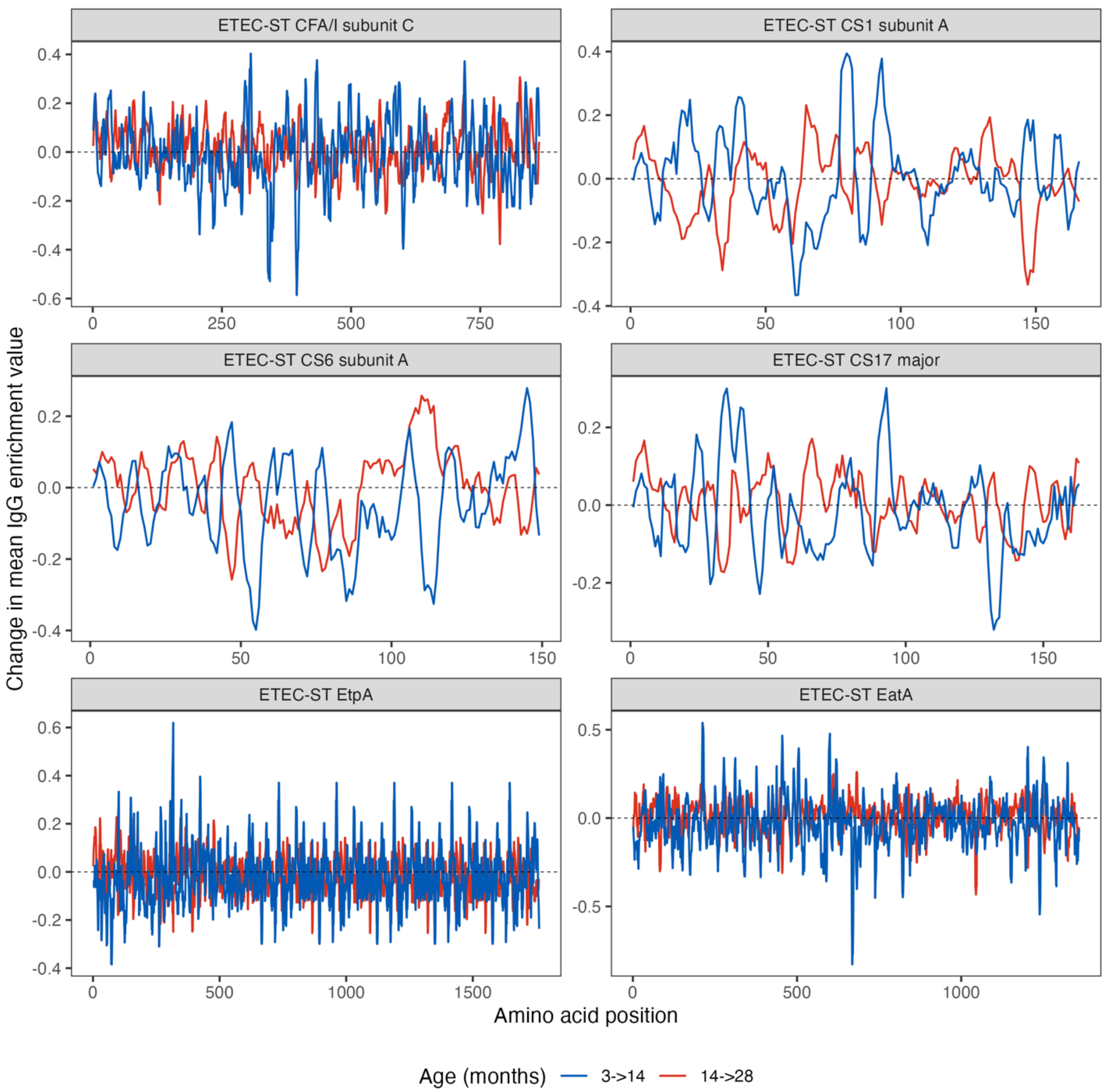

Fig. S16. ETEC-ST magnitude by age and treatment group. Averaged IgG enrichment scores at individual level for a select number of ETEC-ST proteins. For each sample, the IgG enrichment scores were summarized as means by calculating the sum of enrichment values across the protein length and dividing by protein length (number of amino acids per protein). The data summaries are stratified by age and treatment group.

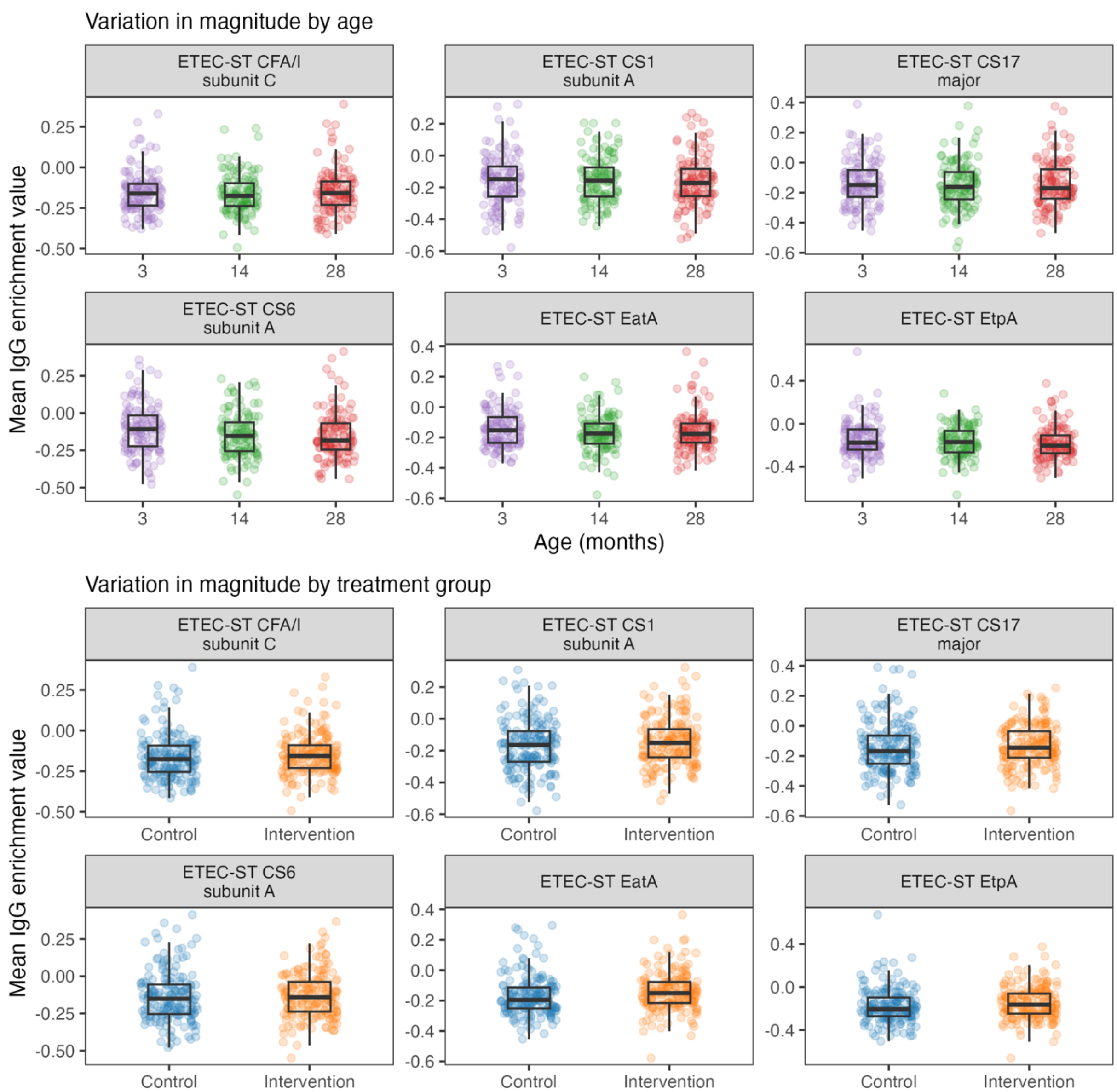

Fig. S17. Measles virus IgG seroprevalence. Protein-level seroprevalence determined as the proportion of children with two or more epitope hits in seroreactive regions in each protein. The data is stratified by treatment group at each sampling visit. The mean seroprevalence and 95% bootstrap confidence interval (1000 bootstrap replicates) are shown.

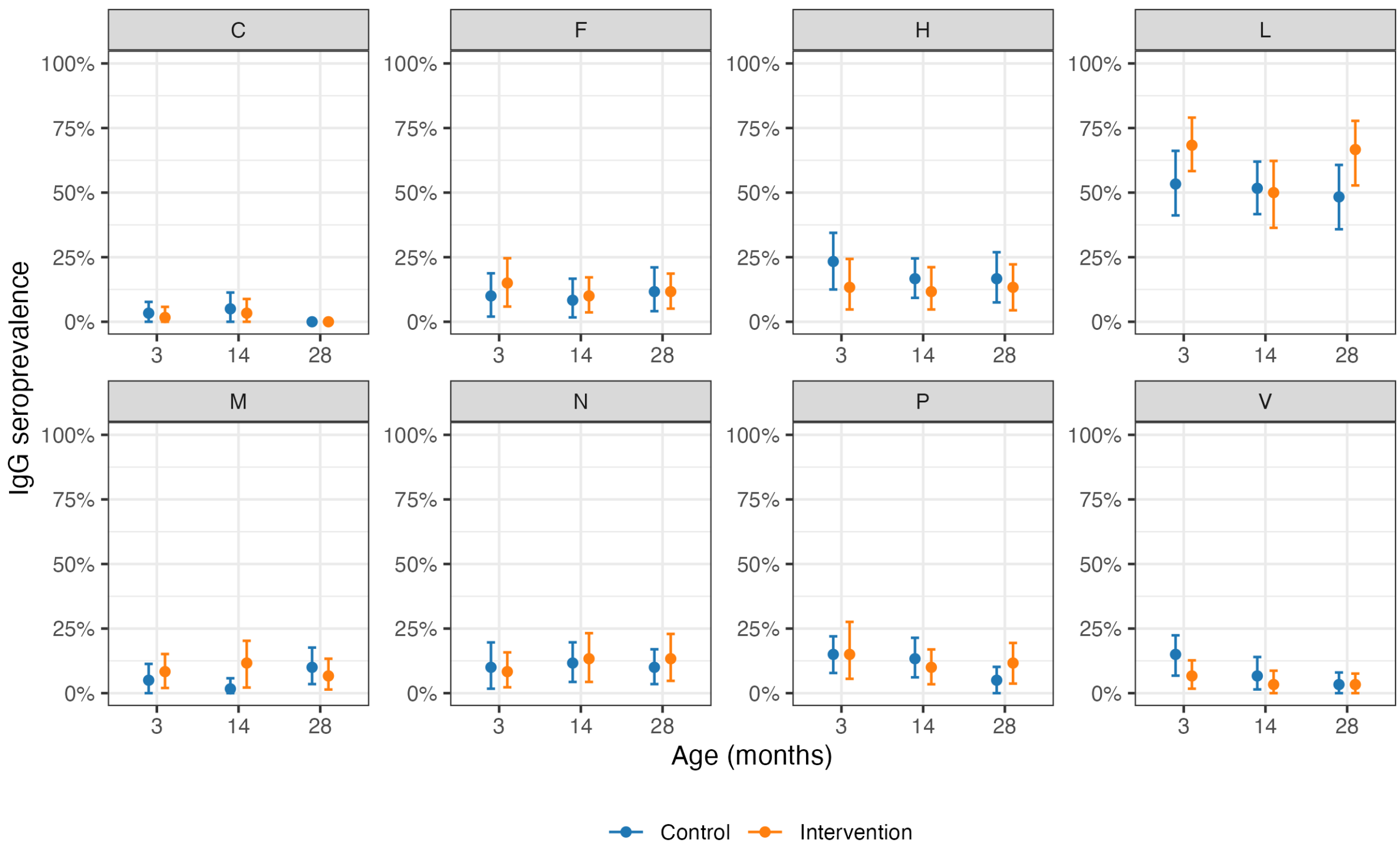

Fig. S18. Comparison of seroprevalence by the serum epitope repertoire analysis (SERA) assay (peptide arrays) and Luminex multiplex bead assay. The comparisons were done for IgG responses to five pathogens measured in both assays.

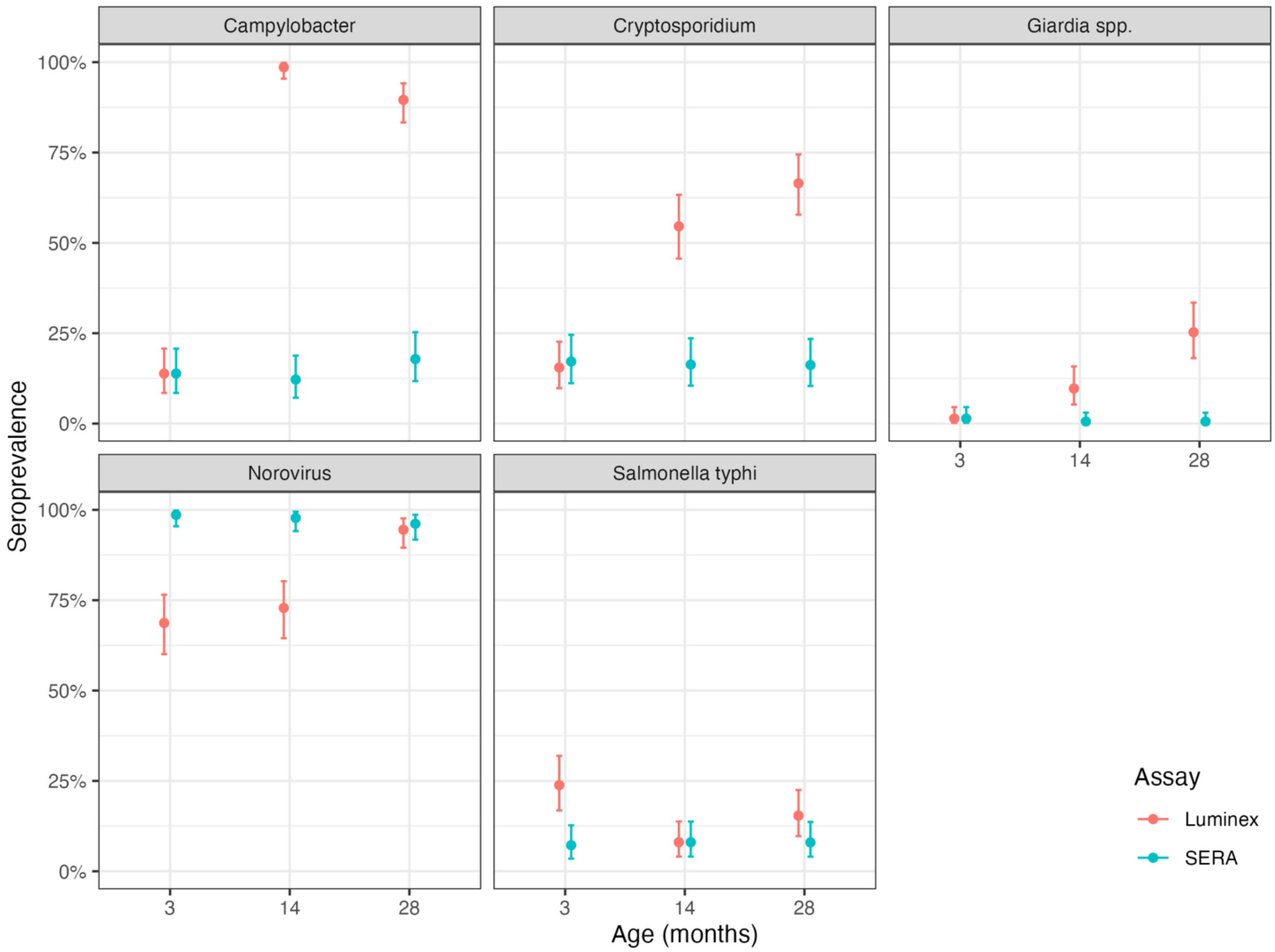

REFERENCES

1. Local Burden of Disease Diarrhoea Collaborators, Mapping geographical inequalities in childhood diarrhoeal morbidity and mortality in low-income and middle-income countries, 2000–17: Analysis for the Global Burden of Disease Study 2017. *Lancet Lond. Engl.* **395**, 1779–1801 (2020).

2. GBD 2017 Causes of Death Collaborators, Global, regional, and national age-sex-specific mortality for 282 causes of death in 195 countries and territories, 1980–2017: A systematic analysis for the Global Burden of Disease Study 2017. *Lancet* **392**, 1736–1788 (2018).

3. GBD 2017 Disease and Injury Incidence and Prevalence Collaborators, Global, regional, and national incidence, prevalence, and years lived with disability for 354 diseases and injuries for 195 countries and territories, 1990–2017: A systematic analysis for the Global Burden of Disease Study 2017. *Lancet Lond. Engl.* **392**, 1789–1858 (2018).

4. K. L. Kotloff, J. P. Nataro, W. C. Blackwelder, D. Nasrin, T. H. Farag, S. Panchalingam, Y. Wu, S. O. Sow, D. Sur, R. F. Breiman, A. S. Faruque, A. K. Zaidi, D. Saha, P. L. Alonso, B. Tamboura, D. Sanogo, U. Onwuchekwa, B. Manna, T. Ramamurthy, S. Kanungo, J. B. Ochieng, R. Omore, J. O. Oundo, A. Hossain, S. K. Das, S. Ahmed, S. Qureshi, F. Quadri, R. A. Adegbola, M. Antonio, M. J. Hossain, A. Akinsola, I. Mandomando, T. Nhampossa, S. Acácio, K. Biswas, C. E. O'Reilly, E. D. Mintz, L. Y. Berkeley, K. Muhsen, H. Sommerfelt, R. M. Robins-Browne, M. M. Levine, Burden and aetiology of diarrhoeal disease in infants and young children in developing countries (the Global Enteric Multicenter Study, GEMS): A prospective, case-control study. *Lancet* **382**, 209–222 (2013).

5. B. F. Arnold, D. L. Martin, J. Juma, H. Mkocha, J. B. Ochieng, G. M. Cooley, R. Omore, E. B. Goodhew, J. F. Morris, V. Costantini, J. Vinjé, P. J. Lammie, J. W. Priest, Enteropathogen antibody dynamics and force of infection among children in low-resource settings. *eLife* **8**, e45594 (2019).

6. K. L. Kotloff, D. Nasrin, W. C. Blackwelder, Y. Wu, T. Farag, S. Panchalingham, S. O. Sow, D. Sur, A. K. M. Zaidi, A. S. G. Faruque, D. Saha, P. L. Alonso, B. Tamboura, D. Sanogo, U. Onwuchekwa, B. Manna, T. Ramamurthy, S. Kanungo, S. Ahmed, S. Qureshi, F. Quadri, A.

Hossain, S. K. Das, M. Antonio, M. J. Hossain, I. Mandomando, S. Acácio, K. Biswas, S. M. Tennant, J. J. Verweij, H. Sommerfelt, J. P. Nataro, R. M. Robins-Browne, M. M. Levine, The incidence, aetiology, and adverse clinical consequences of less severe diarrhoeal episodes among infants and children residing in low-income and middle-income countries: A 12-month case-control study as a follow-on to the Global Enteric Multicenter Study (GEMS). *Lancet Glob. Health* **7**, e568–e584 (2019).

7. A. M. Arzika, R. Maliki, E. B. Goodhew, E. Rogier, J. W. Priest, E. Lebas, K. S. O'Brien, V. Le, C. E. Oldenburg, T. Doan, T. C. Porco, J. D. Keenan, T. M. Lietman, D. L. Martin, B. F. Arnold, Effect of biannual azithromycin distribution on antibody responses to malaria, bacterial, and protozoan pathogens in Niger. *Nat. Commun.* **13**, 976 (2022).

8. C. K. Tiu, F. Zhu, L.-F. Wang, R. de Alwis, Phage ImmunoPrecipitation Sequencing (PhIP-Seq): The promise of high throughput serology. *Pathogens* **11**, 568 (2022).

9. T. Midoro-Horiuti, R. M. Goldblum, Epitope mapping with random phage display library. *Methods Mol. Biol.* **1131**, 477–484 (2014).

10. S. N. Henson, E. A. Elko, P. M. Swiderski, Y. Liang, A. L. Engelbrektson, A. Piña, A. S. Boyle, Z. Fink, S. J. Facista, V. Martinez, F. Rahee, A. Brown, E. J. Kelley, G. A. Nelson, I. Raspet, H. L. Mead, J. A. Altin, J. T. Ladner, PepSeq: A fully in vitro platform for highly multiplexed serology using customizable DNA-barcoded peptide libraries. *Nat. Protoc.* **18**, 396–423 (2023).

11. A. D. Ricci, L. Bracco, E. Salas-Sarduy, J. M. Ramsey, M. S. Nolan, M. K. Lynn, J. Altcheh, G. E. Ballering, F. Torrico, N. Kesper, J. C. Villar, I. S. Marcipar, J. D. Marco, F. Agüero, The trypanosoma cruzi antigen and epitope atlas: Antibody specificities in Chagas disease patients across the Americas. *Nat. Commun.* **14**, 1850 (2023).

12. W. A. Haynes, K. Kamath, J. Bozekowski, E. Baum-Jones, M. Campbell, A. Casanovas-Massana, P. S. Daugherty, C. S. Dela Cruz, A. Dhal, S. F. Farhadian, L. Fitzgibbons, J. Fournier, M. Jhatro, G. Jordan, J. Klein, C. Lucas, D. Kessler, L. L. Luchsinger, B. Martinez, M. Catherine Muenker, L. Pischel, J. Reifert, J. R. Sawyer, R. Waitz, E. A. Wunder, M. Zhang, A. Iwasaki, A. Ko, J. C. Shon, High-resolution epitope mapping and characterization

of SARS-CoV-2 antibodies in large cohorts of subjects with COVID-19. *Commun. Biol.* **4**, 1317 (2021).

13. D. Mohan, D. L. Wansley, B. M. Sie, M. S. Noon, A. N. Baer, U. Laserson, H. B. Larman, PhIP-Seq characterization of serum antibodies using oligonucleotide-encoded peptidomes. *Nat. Protoc.* **13**, 1958–1978 (2018).

14. M. B. Irving, O. Pan, J. K. Scott, Random-peptide libraries and antigen-fragment libraries for epitope mapping and the development of vaccines and diagnostics. *Curr. Opin. Chem. Biol.* **5**, 314–324 (2001).

15. S. P. Luby, M. Rahman, B. F. Arnold, L. Unicomb, S. Ashraf, P. J. Winch, C. P. Stewart, F. Begum, F. Hussain, J. Benjamin-Chung, E. Leontsini, A. M. Naser, S. M. Parvez, A. E. Hubbard, A. Lin, F. A. Nizame, K. Jannat, A. Ercumen, P. K. Ram, K. K. Das, J. Abedin, T. F. Clasen, K. G. Dewey, L. C. Fernald, C. Null, T. Ahmed, J. M. Colford, Effects of water quality, sanitation, handwashing, and nutritional interventions on diarrhoea and child growth in rural Bangladesh: A cluster randomised controlled trial. *Lancet Glob. Health* **6**, e302–e315 (2018).

16. B. F. Arnold, C. Null, S. P. Luby, L. Unicomb, C. P. Stewart, K. G. Dewey, T. Ahmed, S. Ashraf, G. Christensen, T. Clasen, H. N. Dentz, L. C. H. Fernald, R. Haque, A. E. Hubbard, P. Kariger, E. Leontsini, A. Lin, S. M. Njenga, A. J. Pickering, P. K. Ram, F. Tofail, P. J. Winch, J. M. Colford, Cluster-randomised controlled trials of individual and combined water, sanitation, hygiene and nutritional interventions in rural Bangladesh and Kenya: The WASH benefits study design and rationale. *BMJ Open* **3**, e003476 (2013).

17. P. C. Calder, A. C. Carr, A. F. Gombart, M. Eggersdorfer, Optimal nutritional status for a well-functioning immune system is an important factor to protect against viral infections. *Nutrients* **12**, 1181 (2020).

18. F. Pecora, F. Persico, A. Argentiero, C. Neglia, S. Esposito, The role of micronutrients in support of the immune response against viral infections. *Nutrients* **12**, 3198 (2020).

19. A. Lin, S. Ali, B. F. Arnold, M. Z. Rahman, M. Alauddin, J. Grembi, A. N. Mertens, S. L. Famida, S. Akther, M. S. Hossen, P. Mutsuddi, A. K. Shoab, Z. Hussain, M. Rahman, L. Unicomb, S. Ashraf, A. M. Naser, S. M. Parvez, A. Ercumen, J. Benjamin-Chung, R. Haque, T. Ahmed, M. I. Hossain, N. Choudhury, K. Jannat, S. T. Alauddin, S. G. Minchala, R. Cekovic, A. E. Hubbard, C. P. Stewart, K. G. Dewey, J. M. Colford, S. P. Luby, Effects of water, sanitation, handwashing, and nutritional interventions on environmental enteric dysfunction in young children: A cluster-randomized, controlled trial in rural Bangladesh. *Clin. Infect. Dis. Off. Publ. Infect. Dis. Soc. Am.* **70**, 738–747 (2020).

20. J. Reifert, K. Kamath, J. Bozekowski, E. Lis, E. J. Horn, D. Granger, E. S. Theel, J. Shon, J. R. Sawyer, P. S. Daugherty, Serum epitope repertoire analysis enables early detection of lyme disease with improved sensitivity in an expandable multiplex format. *J. Clin. Microbiol.* **59**, e01836-20 (2021).

21. K. Kamath, J. Reifert, T. Johnston, C. Gable, R. J. Pantazes, H. N. Rivera, I. McAuliffe, S. Handali, P. S. Daugherty, Antibody epitope repertoire analysis enables rapid antigen discovery and multiplex serology. *Sci. Rep.* **10**, 5294 (2020).

22. W. A. Haynes, K. Kamath, R. Waitz, P. S. Daugherty, J. C. Shon, Protein-based immunome wide association studies (PIWAS) for the discovery of significant disease-associated antigens. *Front. Immunol.* **12**, 625311 (2021).

23. R. J. Pantazes, J. Reifert, J. Bozekowski, K. N. Ibsen, J. A. Murray, P. S. Daugherty, Identification of disease-specific motifs in the antibody specificity repertoire via next-generation sequencing. *Sci. Rep.* **6**, 30312 (2016).

24. M. L. Paull, T. Johnston, K. N. Ibsen, J. D. Bozekowski, P. S. Daugherty, A general approach for predicting protein epitopes targeted by antibody repertoires using whole proteomes. *PLOS ONE* **14**, e0217668 (2019).

25. J. V. Kringelum, M. Nielsen, S. B. Padkjær, O. Lund, Structural analysis of B-cell epitopes in antibody: Protein complexes. *Mol. Immunol.* **53**, 24–34 (2013).

26. K. F. Sykes, J. B. Legutki, P. Stafford, Immunosignaturing: A critical review. *Trends Biotechnol.* **31**, 45–51 (2013).

27. J. Klein, J. Wood, J. R. Jaycox, R. M. Dhodapkar, P. Lu, J. R. Gehlhausen, A. Tabachnikova, K. Greene, L. Tabacof, A. A. Malik, V. Silva Monteiro, J. Silva, K. Kamath, M. Zhang, A. Dhal, I. M. Ott, G. Valle, M. Peña-Hernández, T. Mao, B. Bhattacharjee, T. Takahashi, C. Lucas, E. Song, D. McCarthy, E. Breyman, J. Tosto-Mancuso, Y. Dai, E. Perotti, K. Akduman, T. J. Tzeng, L. Xu, A. C. Geraghty, M. Monje, I. Yildirim, J. Shon, R. Medzhitov, D. Lutchmansingh, J. D. Possick, N. Kaminski, S. B. Omer, H. M. Krumholz, L. Guan, C. S. Dela Cruz, D. van Dijk, A. M. Ring, D. Putrino, A. Iwasaki, Distinguishing features of long COVID identified through immune profiling. *Nature* **623**, 139–148 (2023).

28. E. Kamau, J. Chen, S. Bajaj, N. Torres, R. Creswell, J. A. Pavlich-Mariscal, C. Donnelly, Z. Cucunubá, B. Lambert, The mathematics of serocatalytic models with applications to public health data. medRxiv [Preprint] (2025). https://doi.org/10.1101/2025.01.21.25320864.

29. M. M. Coughlin, Z. Matson, S. B. Sowers, J. W. Priest, G. P. Smits, F. R. M. van der Klis, A. Mitchell, C. J. Hickman, H. M. Scobie, J. L. Goodson, J. P. Alexander, P. A. Rota, B. Bankamp, Development of a measles and rubella multiplex bead serological assay for assessing population immunity. *J. Clin. Microbiol.* **59**, e02716–20 (2021).

30. M. J. Mina, T. Kula, Y. Leng, M. Li, R. D. de Vries, M. Knip, H. Siljander, M. Rewers, D. F. Choy, M. S. Wilson, H. B. Larman, A. N. Nelson, D. E. Griffin, R. L. de Swart, S. J. Elledge, Measles virus infection diminishes preexisting antibodies that offer protection from other pathogens. *Science* **366**, 599–606 (2019).

31. E. L. Shrock, C. L. Shrock, S. J. Elledge, VirScan: High-throughput profiling of antiviral antibody epitopes. *Bio-Protoc.* **12**, e4464 (2022).

32. A. J. Mentzer, N. Brenner, N. Allen, T. J. Littlejohns, A. Y. Chong, A. Cortes, R. Almond, M. Hill, S. Sheard, G. McVean, U. K. B. Infection Advisory Board, R. Collins, A. V. S. Hill, T. Waterboer, Identification of host-pathogen-disease relationships using a scalable multiplex serology platform in UK Biobank. *Nat. Commun.* **13**, 1818 (2022).

33. J. Rosado, S. Pelleau, C. Cockram, S. H. Merkling, N. Nekkab, C. Demeret, A. Meola, S. Kerneis, B. Terrier, S. Fafi-Kremer, J. de Seze, T. Bruel, F. Dejardin, S. Petres, R. Longley, A. Fontanet, M. Backovic, I. Mueller, M. T. White, Multiplex assays for the identification of serological signatures of SARS-CoV-2 infection: An antibody-based diagnostic and machine learning study. *Lancet Microbe* **2**, e60–e69 (2021).

34. J. A. Grembi, A. Lin, M. A. Karim, M. O. Islam, R. Miah, B. F. Arnold, E. T. R. McQuade, S. Ali, M. Z. Rahman, Z. Hussain, A. K. Shoab, S. L. Famida, M. S. Hossen, P. Mutsuddi, M. Rahman, L. Unicomb, R. Haque, M. Taniuchi, J. Liu, J. A. Platts-Mills, S. P. Holmes, C. P. Stewart, J. Benjamin-Chung, J. M. Colford, E. R. Houpt, S. P. Luby, Effect of water, sanitation, handwashing and nutrition interventions on enteropathogens in children 14 months old: A cluster-randomized controlled trial in rural Bangladesh. *J Infect Dis* **227**, 434–447 (2023).

35. A. Lin, A. Ercumen, J. Benjamin-Chung, B. F. Arnold, S. Das, R. Haque, S. Ashraf, S. M. Parvez, L. Unicomb, M. Rahman, A. E. Hubbard, C. P. Stewart, J. M. Colford, S. P. Luby, Effects of water, sanitation, handwashing, and nutritional interventions on child enteric protozoan infections in rural Bangladesh: A cluster-randomized controlled trial. *Clin. Infect. Dis. Off. Publ. Infect. Dis. Soc. Am.* **67**, 1515–1522 (2018).

36. A. Lin, A. N. Mertens, S. Tan, M. Z. Rahman, L. Hester, L. Kim, B. F. Arnold, M. R. Karim, S. Shahriar, S. Ali, A. K. Shoab, M. S. Hossen, P. Mutsuddi, S. L. Famida, S. Akther, M. Rahman, L. Unicomb, Z. Butzin-Dozier, C. Hemlock, A. E. Hubbard, C. P. Stewart, L. C. H. Fernald, J. M. Colford, S. P. Luby, F. S. Dhabhar, Effects of drinking water, sanitation, handwashing, and nutritional interventions on immune status in young children: A cluster-randomized controlled trial in rural Bangladesh. medRxiv [Preprint] (2021). https://doi.org/10.1101/2021.11.10.21266206.

37. J. E. Ludert, M. C. Ruiz, C. Hidalgo, F. Liprandi, Antibodies to rotavirus outer capsid glycoprotein VP7 neutralize infectivity by inhibiting virion decapsidation. *J. Virol.* **76**, 6643–6651 (2002).

38. Y. Hoshino, K. Nishikawa, D. A. Benfield, M. Gorziglia, Mapping of antigenic sites involved in serotype-cross-reactive neutralization on group A rotavirus outercapsid glycoprotein VP7. *Virology* **199**, 233–237 (1994).

39. K. Y. Green, A. Z. Kapikian, Identification of VP7 epitopes associated with protection against human rotavirus illness or shedding in volunteers. *J. Virol.* **66**, 548–553 (1992).

40. P. Premkumar, B. Lopman, S. Ramani, A. Paul, B. Gladstone, J. Muliyil, I. Mukhopadhya, U. Parashar, G. Kang, Association of serum antibodies with protection against rotavirus infection and disease in South Indian children. *Vaccine* **32**, A55–A61 (2014).

41. J. Mandolo, L. Mulira, M. Moyo, M. Mvula, F. Mtonga, M. Y. R. Henrion, W. Wotcheni, N. A. Cunliffe, K. G. Barnes, K. C. Jambo, K. C. Jere, Maternal-infant rotavirus-specific antibody kinetics to inform timing of vaccine boosting in Malawi: An observational study. *PLoS Med.* **22**, e1004734 (2025).

42. M. Tahara, J.-P. Bürckert, K. Kanou, K. Maenaka, C. P. Muller, M. Takeda, Measles virus hemagglutinin protein epitopes: The basis of antigenic stability. *Viruses* **8**, 216 (2016).

43. C. P. Campillay-Véliz, J. J. Carvajal, A. M. Avellaneda, D. Escobar, C. Covián, A. M. Kalergis, M. K. Lay, Human norovirus proteins: Implications in the replicative cycle, pathogenesis, and the host immune response. *Front. Immunol.* **11**, 961 (2020).

44. N. A. Vielot, A. Brinkman, C. DeMaso, S. Vilchez, L. C. Lindesmith, F. Bucardo, Y. Reyes, R. S. Baric, E. P. Ryan, R. Braun, S. Becker-Dreps, Breadth and dynamics of human norovirus-specific antibodies in the first year of life. *J. Pediatr. Infect. Dis. Soc.* **11**, 463–466 (2022).

45. R. M. Zinkernagel, Maternal antibodies, childhood infections, and autoimmune diseases. *N. Engl. J. Med.* **345**, 1331–1335 (2001).

46. J. Zhou, Y. Zhou, K. Luo, Q. Liao, W. Zheng, H. Gong, H. Shi, S. Zhao, K. Wang, Q. Qiu, B. Dai, L. Ren, L. Wang, L. Gao, M. Xu, N. Liu, W. Lu, N. Zheng, X. Chen, Z. Chen, J. Yang, S. Cauchemez, H. Yu, The transfer of maternal antibodies and dynamics of maternal and

natural infection-induced antibodies against coxsackievirus A16 in Chinese children 0–13 years of age: A longitudinal cohort study. *BMC Med.* **20**, 436 (2022).

47. X. Wei, J. Yang, L. Gao, L. Wang, Q. Liao, Q. Qiu, K. Luo, S. Yu, Y. Zhou, F. Liu, Q. Chen, J. Zhang, B. Dai, H. Yang, J. Zhou, W. Xing, X. Chen, M. He, L. Ren, J. Guo, L. Luo, P. Wu, Z. Chen, H. R. van Doorn, S. Cauchemez, B. J. Cowling, H. Yu, The transfer and decay of maternal antibodies against enterovirus A71, and dynamics of antibodies due to later natural infections in Chinese infants: A longitudinal, paired mother-neonate cohort study. *Lancet Infect. Dis.* **21**, 418–426 (2021).

48. V. Blazevic, M. Malm, H. Honkanen, M. Knip, H. Hyöty, T. Vesikari, Development and maturation of norovirus antibodies in childhood. *Microbes Infect.* **18**, 263–269 (2016).

49. S. J. Carmona, M. Nielsen, C. Schafer-Nielsen, J. Mucci, J. Altcheh, V. Balouz, V. Tekiel, A. C. Frasch, O. Campetella, C. A. Buscaglia, F. Agüero, Towards high-throughput immunomics for infectious diseases: Use of next-generation peptide microarrays for rapid discovery and mapping of antigenic determinants. *Mol. Cell. Proteomics* **14**, 1871–1884 (2015).

50. G. J. Xu, T. Kula, Q. Xu, M. Z. Li, S. D. Vernon, T. Ndung'u, K. Ruxrungtham, J. Sanchez, C. Brander, R. T. Chung, K. C. O'Connor, B. Walker, H. B. Larman, S. J. Elledge, Comprehensive serological profiling of human populations using a synthetic human virome. *Science* **348**, aaa0698 (2015).

51. E. J. Kelley, S. N. Henson, F. Rahee, A. S. Boyle, A. L. Engelbrektson, G. A. Nelson, H. L. Mead, N. L. Anderson, M. Razavi, R. Yip, J. T. Ladner, T. J. Scriba, J. A. Altin, Virome-wide detection of natural infection events and the associated antibody dynamics using longitudinal highly-multiplexed serology. *Nat. Commun.* **14**, 1783 (2023).

52. J. W. Priest, D. M. Moss, Measuring *Cryptosporidium* serologic responses by multiplex bead assay. *Methods Mol. Biol.* **2052**, 61–85 (2020).

53. Y. Chan, K. Fornace, L. Wu, B. F. Arnold, J. W. Priest, D. L. Martin, M. A. Chang, J. Cook, G. Stresman, C. Drakeley, Determining seropositivity-A review of approaches to define

population seroprevalence when using multiplex bead assays to assess burden of tropical diseases. *PLoS Negl. Trop. Dis.* **15**, e0009457 (2021).

54. S. Dhakal, M. J. Karim, A. Al Kawsar, J. Irish, M. Rahman, C. Tupps, A. Kabir, R. Imtiaz, Post-intervention epidemiology of STH in Bangladesh: Data to sustain the gains. *PLoS Negl. Trop. Dis.* **14**, e0008597 (2020).

55. A. S. Azman, S. A. Lauer, T. R. Bhuiyan, F. J. Luquero, D. T. Leung, S. T. Hegde, J. B. Harris, K. K. Paul, F. Khaton, J. Ferdous, J. Lessler, H. Salje, F. Qadri, E. S. Gurley, *Vibrio cholerae* O1 transmission in Bangladesh: Insights from a nationally representative serosurvey. *Lancet Microbe* **1**, e336–e343 (2020).

56. P. Korpe, Z. Ni, M. Kabir, M. Alam, T. Ferdous, R. Ara, R. M. Munday, R. Haque, P. Duggal, Prospective cohort study of *Cryptosporidium* infection and shedding in infants and their households. *Clin. Infect. Dis.* **76**, 2178–2186 (2023).

57. J. J. Sanchez, M. A. Alam, C. B. Stride, M. A. Haque, S. Das, M. Mahfuz, D. E. Roth, P. D. Sly, K. Z. Long, T. Ahmed, *Campylobacter* infection and household factors are associated with childhood growth in urban Bangladesh: An analysis of the MAL-ED study. *PLoS Negl. Trop. Dis.* **14**, e0008328 (2020).

58. C. Amour, J. Gratz, E. Mduma, E. Svensen, E. T. Rogawski, M. McGrath, J. C. Seidman, B. J. J. McCormick, S. Shrestha, A. Samie, M. Mahfuz, S. Qureshi, A. Hotwani, S. Babji, D. R. Trigoso, A. A. M. Lima, L. Bodhidatta, P. Bessong, T. Ahmed, S. Shakoor, G. Kang, M. Kosek, R. L. Guerrant, D. Lang, M. Gottlieb, E. R. Houpt, J. A. Platts-Mills, Etiology, risk factors, and interactions of enteric infections and malnutrition and the consequences for child health and development project (MAL-ED) network investigators, epidemiology and impact of *Campylobacter* infection in children in 8 low-resource settings: Results from the MAL-ED study. *Clin. Infect. Dis.* **63**, 1171–1179 (2016).

59. G. Lee, W. Pan, P. Peñataro Yori, M. Paredes Olortegui, D. Tilley, M. Gregory, R. Oberhelman, R. Burga, C. B. Chavez, M. Kosek, Symptomatic and asymptomatic *Campylobacter* infections associated with reduced growth in Peruvian children. *PLoS Negl. Trop. Dis.* **7**, e2036 (2013).

60. A. Albutti, Rational design of a multi epitope vaccine against *Salmonella typhi* via subtractive proteomics, reverse vaccinology and molecular modeling. *Sci. Rep.* **15**, 32057 (2025).
